# Large‐Scale Production of Transfusion‐Ready Red Blood Cells From Induced Pluripotent Stem Cells

**DOI:** 10.1002/advs.202504725

**Published:** 2025-07-12

**Authors:** Eszter Varga, Eelke Brandsma, Brenda E. Juarez‐Garza, Renuka P. E. Ramlal, Julien J. Karrich, Adrien Laurent, Athina Chavli, Ruthmila Paskel, Kerly Fu, Richard A. Flavell, Marieke von Lindern, Derk Amsen, Marieke E. Klijn, Emile van den Akker

**Affiliations:** ^1^ Department of Hematopoiesis Sanquin Research Amsterdam Amsterdam 1066CX The Netherlands; ^2^ Department of Biotechnology Delft University of Technology Delft 2629HZ The Netherlands; ^3^ Department of Immunobiology Yale University School of Medicine New Haven CT 06520 USA; ^4^ Howard Hughes Medical Institute Yale University School of Medicine New Haven CT 06520 USA

**Keywords:** cellular therapy, hematopoietic, iPSC, large‐scale, organoid, red blood cells, transfusion

## Abstract

There is a constant worldwide need for blood products, traditionally obtained from donations. In vitro red blood cell (RBC) production could supplement this demand and offer benefits such as thorough screening for improved safety, the possibility of genetic manipulation to restore genetic deficiencies, and therapeutic loading. Induced pluripotent stem cells (iPSCs) are a promising cell source for transfusable RBCs due to their immortality and independence from donors. However, current iPSC differentiation protocols—including both monolayer and embryoid body‐based systems—have failed to produce sufficient erythroid cells (10^11–12^ per unit) for therapeutic application, primarily due to developmental immaturity, inefficient enucleation (5–25%), and suboptimal, static culture conditions lacking physiological relevance. This study describes the optimization of an iPSC to RBC differentiation platform and its step‐by‐step translation process to dynamic culture conditions, allowing scalability and eventual bioreactor application. The optimized dynamic culture yields ≈4.6 × 10^3^ RBC/iPSC, requiring an estimated ≈4.9 × 10^7^ iPSCs to produce a minitransfusion unit, achieving a consistent 40–70% enucleation rate and bona fide function, demonstrated by both in vitro and in vivo assays. Our feeder‐free, GMP‐compatible system accomplishes an enucleated RBC production rate sufficient for large‐scale application and serves as a bridge to large‐scale bioreactor RBC production, facilitating clinical application.

## Graphical abstract

A scalable, dynamic culture system is developed to produce red blood cells from induced pluripotent stem cells. It achieves high yields with up to 70% enucleation, not previously shown in dynamic systems, overcoming key limitations and enabling large‐scale production. This marks an important step toward lab‐grown red blood cells to help address the growing global need for reliable transfusion products.

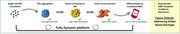

## 1 Introduction

There is a constant worldwide need for blood products, traditionally obtained from blood donations. In vitro RBC production could supplement this demand and offer benefits such as the ability to thoroughly screen products for improved safety, the possibility of genetic manipulation to restore genetic deficiencies, and therapeutic loading. This need is further complicated by the critical challenges posed by rare blood group phenotypes, which require careful identification and management in transfusion medicine to reduce alloimmunization risks. Ensuring safe and sufficient blood supplies is particularly important for patients across diverse clinical settings and ethnic backgrounds—including those with chronic transfusion needs (e.g., sickle cell disease, thalassemia) and acute situations such as trauma or surgery. Rare phenotypes are associated with limited donor availability due to uncommon antigenic expressions, complicating compatibility and increasing the risk of adverse events. In 2018, over 6500 transfusion‐related adverse events were reported globally, highlighting the urgency of improving blood product safety and availability.^[^ ^1^ ^]^ Current reliance on donor‐derived blood components remains insufficient to address these challenges. In vitro‐derived blood components represent a promising avenue to meet global demands while addressing alloimmunization risks. These components offer advantages such as improved safety, enhanced monitoring, and customizable transfusion products. RBCs, the most commonly transfused blood component, represent a particularly important target, with global usage estimated at 28.8 units per 1000 population in 2018.^[^ ^1^ ^]^ The in vitro production of RBCs with rare phenotypes could not only meet the current demand but also support novel applications like drug delivery, genetic disorder treatments, and immune modulation. In vitro derivation provides the possibility to in vitro modify at the stem cell/progenitor stage, followed by differentiation into RBCs, which can be used to treat genetic disorders or in combination with cargo‐loading to target specific pathologies (e.g., cancer, alloimmunization, autoimmune disease, enzyme deficiency).^[^ ^2^, ^3^, ^4^ ^]^ Functional, cultured RBCs (cRBCs) have been successfully derived from primary hematopoietic stem cell (HSC) sources and demonstrated safety in an autologous proof‐of‐concept study (ISRCTN42886452).^[^ ^5^, ^6^, ^7^, ^8^, ^9^, ^10^ ^]^ However, current sources, such as cord blood (CB) or peripheral mononuclear cells (PBMCs), remain limited by donor dependency. An alternative, immortal source such as iPSCs holds great promise.^[^ ^11^, ^12^ ^]^ iPSCs represent an excellent, clinically relevant source as they are i) produced from somatic material, negating the ethical constraints associated with embryonic stem cells (ESCs), ii) immortal, iii) capable of generating all intra‐embryonic cell types, iv) applicable as universal or immunologically catered cell lines, and v) can be generated in a transgene‐free, GMP‐compliant manner. However, in vitro differentiation of iPSCs into developmentally mature hematopoietic effector cells (e.g., enucleated RBCs, high‐ploidy megakaryocytes) has proven to be a significant challenge.^[^ ^13^, ^14^, ^15^, ^16^ ^]^ The fetal‐like phenotype commonly observed in iPSC‐derived RBCs—marked by elevated fetal hemoglobin (HbF) and increased oxygen affinity—may be suitable as conventional transfusion products based on evidence from naturally occurring and clinically utilized HbF‐expressing RBCs.^[^ ^17^, ^18^, ^19^ ^]^ Moreover, they might offer particular advantages for preterm infants, where HbF‐RBC transfusions could reduce adverse events.^[^ ^20^ ^]^ Human hematopoietic development is characterized by independent spatio‐temporal hematopoietic waves, categorized based on the organ of origin: 1) yolk sac, 2) fetal liver, 3) bone marrow or the progenitor of origin: 1) hemangioblast in yolk sac, 2) erythro‐myeloid progenitors (EMPs), initially in yolk sac and later migrating to fetal liver and 3) HSC arising in the aorta gonad mesonephros (AGM)‐region, first repopulating the fetal liver and later the bone marrow. While iPSC differentiation protocols replicate certain aspects of hematopoietic development, current state‐of‐the‐art methods frequently produce developmentally immature hematopoietic cells, likely originating from early progenitors rather than bona fide HSCs.^[^ ^21^, ^22^, ^23^ ^]^ Further research is necessary to generate iPSC‐derived RBCs with developmentally mature effector characteristics suitable for transfusion. Multiple methods have been employed to differentiate ESCs/iPSC into the erythroid lineage (Table S1, Supporting Information) and primarily used in erythroid developmental studies (both healthy or affected) and disease modeling. However, all these methods are insufficient for production of transfusion‐ready RBCs, mainly due to their high embryonic globin content, inefficient enucleation, and low yield. Particularly, when starting from iPSCs, the reported yields of enucleated cells have remained below 25%, often relying on mouse feeder cells in the final step of differentiation, negating clinical applications (Table S1, Supporting Information). Recently, Bernecker et al. reported a system that initiates a specific microenvironment compatible with increased enucleation potential (40–60%), and requires minimal growth factor addition.^[^ ^24^ ^]^ Unlike previous reports, this is the only system in which spontaneous differentiation applied during the initiation step avoids specifically directing cells toward the mesodermal lineage (Table S1, Supporting Information). Spontaneous embryoid body (EB) formation, however, allows differentiation into multiple germ layers, including the mesodermal lineage. Consequently, a fraction of the formed EBs has the potential to generate hematopoietic organoids (HeOs), providing a specific microenvironment that can support the in vitro emergence of enucleation‐proficient, hematopoietic progeny. This system, based on static‐ and surface‐intensive culturing, is thus applicable for small‐scale RBC production. However, proof‐of‐principle Phase I trials require mini‐transfusion units containing 10^10–11^ cRBCs, whereas actual transfusion units contain ≈1–2 × 10^12^ RBCs. Scaling up production to this magnitude may be feasible through stirred bioreactors. Some aspects of this transition are already feasible, as recent advances in 3D‐iPSC maintenance using shake flasks or bioreactors have eliminated the need for traditional 2D culture steps, enabling scalable iPSC expansion. However, successful scale‐up requires the entire differentiation workflow to be adaptable to dynamic suspension culturing. One group successfully carried out the entire iPSC‐RBC (iRBC) differentiation in dynamic culture environments, allowing large‐scale culturing, but their enucleation rate in feeder‐free cultures remained at 6%, ultimately resulting in inadequate yields of transfusion‐ready RBCs.^[^ ^25^, ^26^ ^]^

Here we present a comprehensive improvement and translation of the entire differentiation platform described by Bernecker et. al in 2019, transitioning from static/adherent to dynamic/suspension culture conditions.^[^ ^24^ ^]^ This translation not only enables scalability but also paves the way for future applications in bioreactors. Addressing the challenges of variability in EB content, size and shape, which can affect the reproducibility of HeO formation, we have optimized the EB‐ and HeO‐formation phases, resulting in highly uniform cell structures and offering a reproducible differentiation workflow. Furthermore, the dynamic translation process described here is entirely feeder‐free, xeno‐free, and GMP‐compatible, bridging small‐scale static culturing to large‐scale bioreactor‐based RBC production, thus advancing clinical transfusion application.

## 2 Results

### 2.1 iPSC to Erythroid Differentiation Resembling Distinct Developmental Maturity

**Figure 1:**
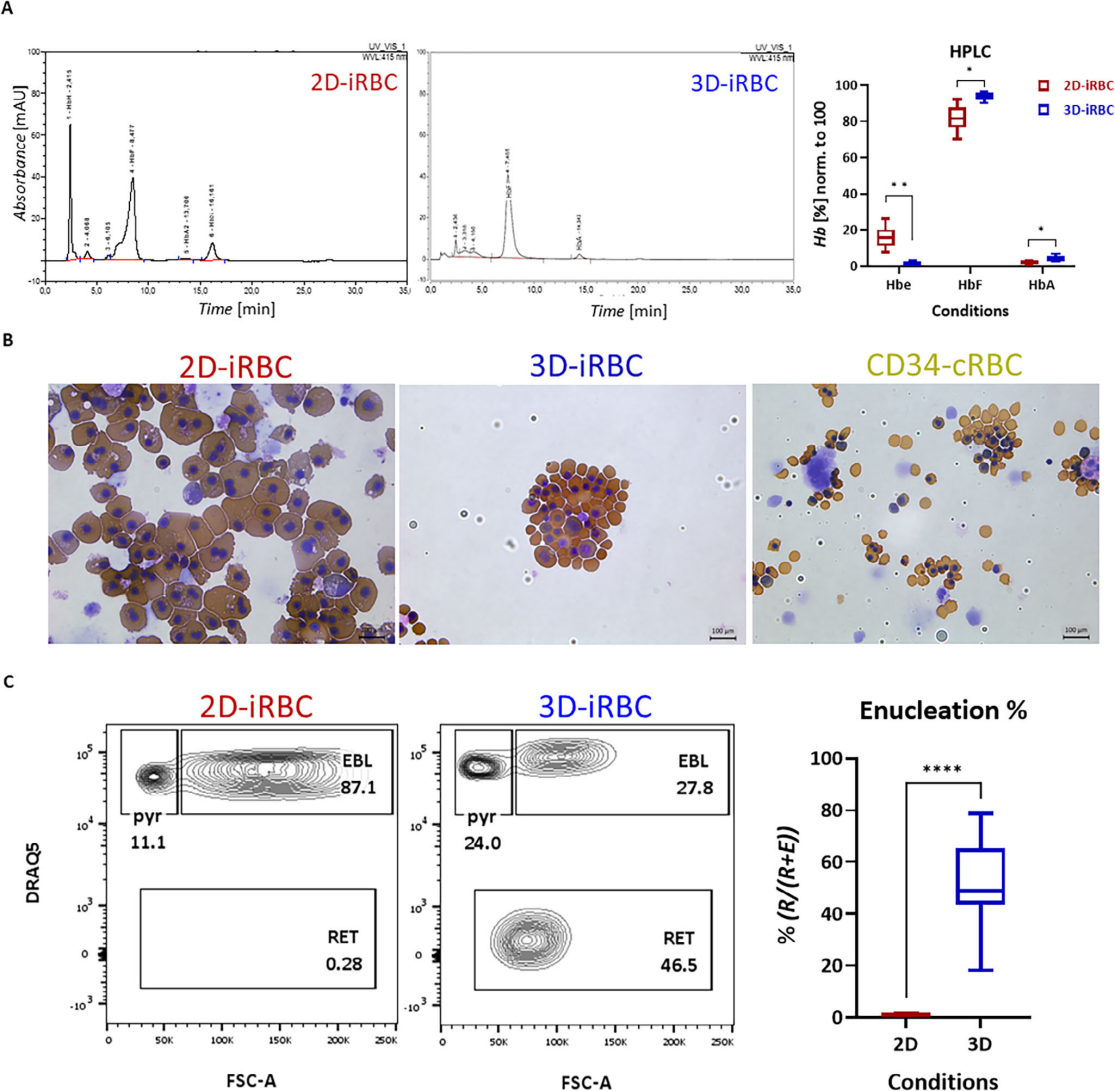
Derivation of developmentally more mature erythroid cells. A) HPLC track (left) of 2D‐iRBCs and 3D‐iRBCs; quantification of Hbe (α2ε2), HbF (α2γ2), HbA (α2β2) based on HPLC tracks (right), *n*≥5, unpaired t test with Welch, Holm–Šídák method, Alpha = 0.05. B) Cytospins combined with benzidine‐giemsa staining. C) Example flow cytometry plots of CD235+ populations depicting DRAQ5‐ RET%, DRAQ5+ pyrenocytes%, and EBL% (left); enucleation% based on flow cytometry calculated by RET% / (RET% + EBL%) (right), *n* ≥ 8, unpaired t test with Welch, 95% CI.

It has been previously shown by us that iRBCs can be differentiated from iPSCs using a monolayer (2D) differentiation system based on growth factor‐dependent hematopoietic initiation.^[^ ^27^ ^]^ This system gives rise to hemoglobinized iRBCs expressing both fetal hemoglobin (α2γ2) and significant levels of embryonic globins (α2ε2) as shown by high‐performance liquid chromatography (HPLC) (**Figure** **1**A). The nucleated 2D‐iRBCs were larger in size compared to their definitive counterparts (CD34‐derived) demonstrated by cytospin combined with giemsa‐benzidine staining (Figure 1B). In addition, the 2D‐derived iRBCs exhibited enucleation deficiency (<5% enucleation) both in feeder‐free conditions (Figure 1C), and on feeder layers (Figure S1A, Supporting Information). These characteristics suggest that the 2D‐iRBCs resemble a developmentally immature, primitive wave of erythropoiesis, and due to enucleation deficiency, the system may not be suitable for transfusion. The erythroid cells derived from the 2D‐iRBC system were compared to those produced by another protocol based on 3D‐iPSC differentiation initiation, through growth factor‐independent spontaneous EB formation, followed by enucleation‐proficient HeO formation.^[^ ^24^ ^]^ The iRBCs derived using this method were hemoglobinized (Figure 1A,B) and primarily expressed fetal hemoglobin (α2γ2) with a near absence of embryonic globins (α2ε2) (Figure 1A). The size of the 3D‐iRBCs was smaller than their 2D counterparts (Figure 1B). Notably, the enucleation rate of 3D‐iRBC cells was consistently 44–65%, tested on three different iPSC lines, albeit with some line‐to‐line differences (Figure 1C; Figure S1B, Supporting Information). Based on these characteristics (size, globin type, enucleation potential), the 3D‐iRBC cells resembled a developmentally more mature, fetal wave of hematopoiesis.

We conclude that the 2D‐iRBC differentiation system is applicable in fundamental research but unsuitable to generate RBCs for transfusion.^[^ ^27^ ^]^ On the other hand, the 3D‐iRBC differentiation described above yields erythroid cells that are fetal in nature and, importantly, capable of significant enucleation and thus a promising protocol for future transfusion applications.^[^ ^24^ ^]^

### 2.2 Optimized EB Formation in Dynamic Culture Enhances HeO Formation

The 3D‐iRBC differentiation system comprises three Phases (Phs): I) EB formation, II) HeO formation, and III) erythroid differentiation (**Figure** **2**A). The HeOs are arising from spontaneously formed EBs. Within ≈3–4 weeks, the formed organoids (Figure 2B) become proficient at producing hematopoietic stem and progenitor cells (HSPCs), allowing for multiple harvests from the same culture. However, only an average of 9% of EBs progressed to hematopoietic‐proficient HeOs (Figure 2B), highlighting a bottleneck in scaling up due to the limited conversion efficiency. Additionally, the collagenase IV treatment of the iPSC colonies (day 0) involves a cumbersome manual procedure, in which the entire colony must be lifted from the surface after 1–1.5 h of incubation, making it impractical for large‐scale inoculation. This treatment also results in uneven iPSC clump sizes, leading to heterogeneously sized EBs, which adversely affects reproducibility. To overcome these limitations, we aimed to improve overall efficiency. Instead of collagenase IV treatment, iPSCs are treated with accumax, passed through on a 40 µm strainer to ensure single‐cell suspension. iPSC aggregates are generated by inoculating 0.3–0.6 × 10^6^ mL single cells into Low‐attachment (LA) plates placed on an orbital shaker (55 rpm), following a conventional 3D‐iPSC suspension culturing procedure. At this stage, iPSC aggregates are cultured with Bfgf‐containing media. Two to five days post‐inoculation, the iPSC aggregates are directly converted into EBs (without dissociation) by culturing in media without bFGF for 5 days (day 0–5) under continuous shaking. Compared to the original method, the derived EB are more regularly shaped (56% versus 6%) and smaller but exhibit greater homogeneity in size (Figure 2C; Figure S2A, Supporting Information). The single cell/dynamic culturing results in an average of 745 (±453) EB mL^−1^, whereas the original system is unquantifiable due to varying size and cell content within the clump input. Importantly, these optimization steps increased the average HeO forming potential per EB to av. 60% (Figure 2B), without affecting the weekly HSPC production efficiency per HeO (Figure 2D). To investigate factors influencing erythroid‐lineage proficiency, EB size was assessed in relation to HeO formation and HPSC output using individually plated EBs. In the original system, EBs capable of forming HeOs were significantly larger than the EBs unable to form HeOs. However, this trend was not observed in the optimized Phase I system (Figure S2B, Supporting Information). Furthermore, EB size did not differ significantly between erythroid‐proficient and nonerythroid‐proficient HeOs in either system. Interestingly, HeOs derived from the original system were associated with significantly larger EBs compared to those from the optimized protocol (Figure S2C, Supporting Information), rendering the correlation between EB size and proficiency inconclusive. The optimization did not alter the expression of CD34, CD43, CD45 (hematopoietic), and CD235 (erythroid) markers over the 4‐week period, although higher CD34 expression was observed with the original method at the 6‐week timepoint (Figure 2E; Figure S2D, Supporting Information). The average fold expansion of the produced HSPCs in erythroid expansion/differentiation media was, on average 56‐fold, which was comparable to the original method (Figure 2F). The enucleation rate was also not affected by the optimization procedure and remained within the 50–63% range, quantified by flow cytometry (Figure 2G).

Figure 2Optimized EB formation in dynamic culture conditions enhances HeO formation. A) Timeline of the 3D‐iRBC differentiation system; 1: original platform; 2: optimized platform. B) HeO morphology (left); quantification of the HeO forming potential/EB (right), *n *≥ 5, unpaired Mann‐Whitney test, 95% CI. C) Quantification of round or irregular EB shape, *n *≥ 36 (left) and size (right) in relation with static versus dynamic EB culturing, *n *≥ 36, unpaired t test with Welch, 95% RI. D) Weekly HSPC production efficiency, *n *≥ 4, unpaired Mann‐Whitney test, Holm‐Šídák method, Alpha = 0.05. E) Weekly marker expression pattern of the HSPCs using flow cytometry, *n *≥ 3, unpaired Mann‐Whitney test, Holm‐Šídák method, Alpha = 0.05. F) Expansion potential of the produced HSPCs during erythroid terminal differentiation. The quantifications are shown with mean and SEM, *n* ≥ 7, Mixed‐effect with Geisser‐Greenhouse, Ṧidak's comparison, Alpha = 0.05, 95% CI. G) Enucleation% of the iRBCs, based on flow cytometry using CD235/CD71 and DRAQ5 staining, *n *≥ 17, unpaired Mann‐Whitney test, 95% CI.

**Figure:**
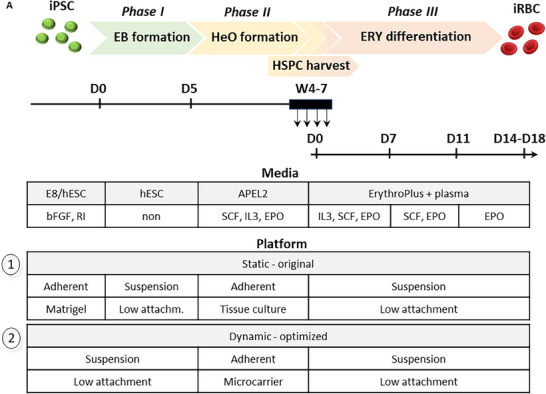

**Figure:**
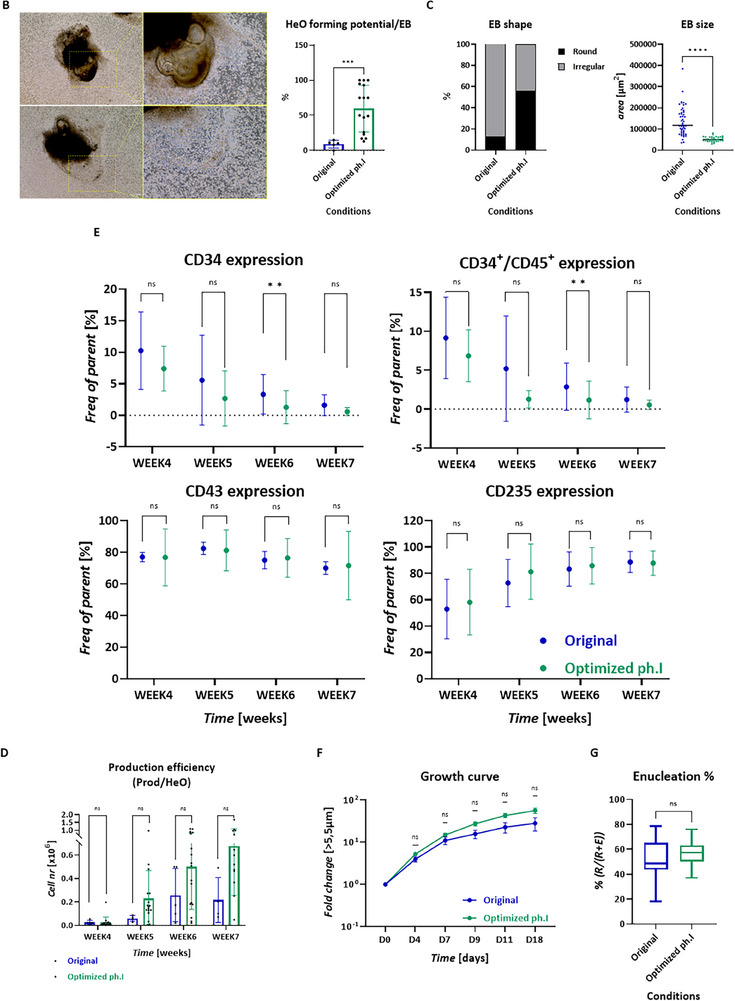

We thus successfully transitioned to a single‐cell/dynamic EB platform, replacing iPSC clump inoculation and static culturing. This approach ensures homogeneous media mixing, consistent EB quality and quantity, and is less labor‐intensive. The unique approach of direct transition from single‐cell derived iPSC aggregates to EBs offers a more controlled and adaptable system that could support long‐term 3D‐iPSC maintenance. Importantly, these improvements significantly enhanced HeO formation efficiency without negatively affecting downstream hematopoietic or erythroid differentiation.

### 2.3 Microcarriers Provide Adequate Surface for Efficient HeO Formation Under Dynamic Conditions

**Figure 3:**
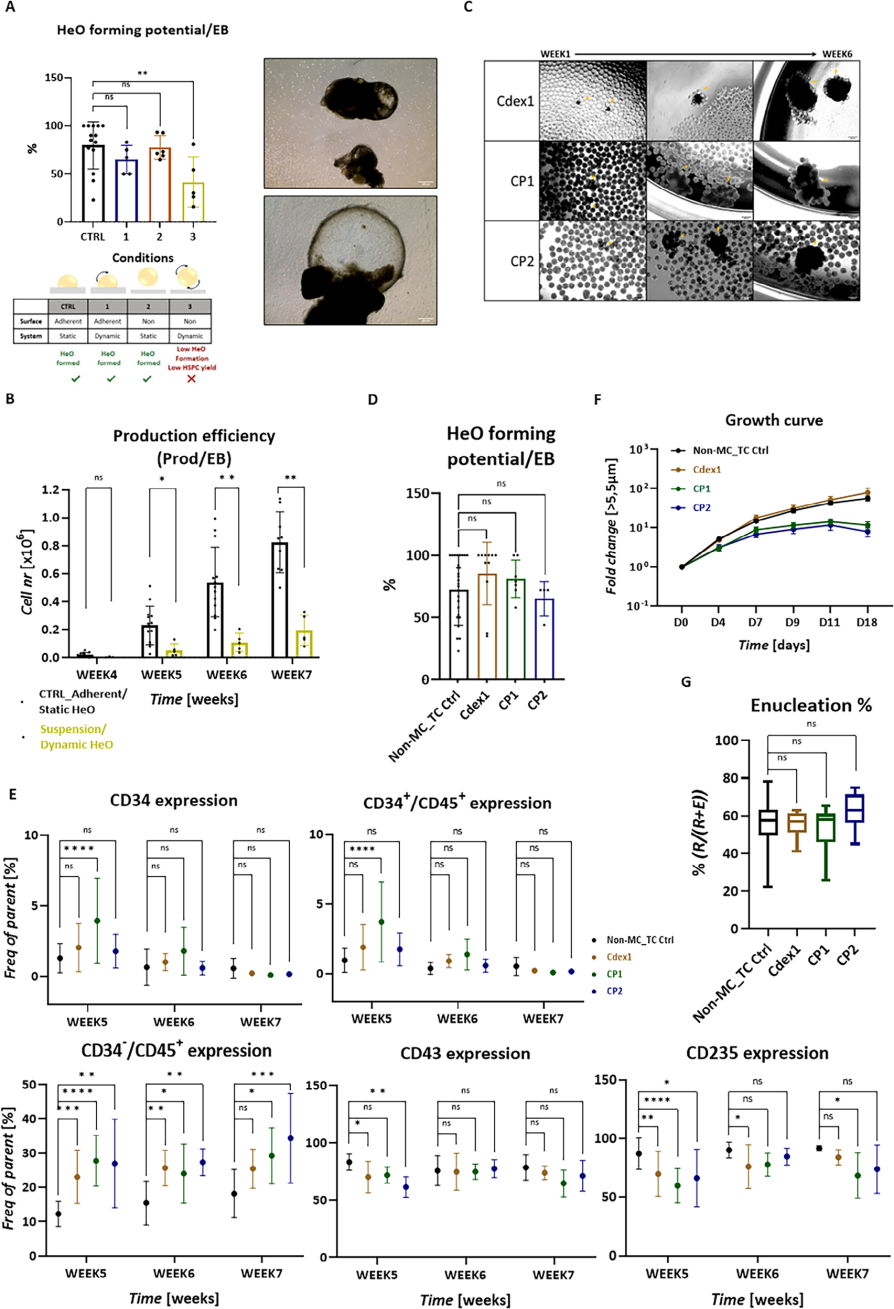
Erythroid proficient HeO formation under dynamic culture condition. A) HeO formation potential (left), *n *≥ 5, Dunnett's multiple comparison, Alpha = 0.05, 95% CI; morphology of HeOs on non‐adherent surface (right). B) Weekly HSPC production efficiency, *n *≥ 3, unpaired Mann‐Whitney test, Holm–Šídák method, Alpha = 0.05. C) Morphology of MC‐derived complexes using 3 types of MCs. D) HeO forming potential on MC surface, *n* ≥ 11, unpaired Mann‐Whitney test, Holm‐Šídák method, Alpha = 0.05. E) Weekly marker expression pattern of HSCPs derived from MC‐HeOs, *n *≥ 4, Dunnett's multiple comparison, Alpha = 0.05, 95% CI. F) Expansion potential of the MC‐HeO produced HSPCs during erythroid terminal differentiation. The quantifications are shown with mean and SEM, *n *≥ 8, Mixed‐effect with Geisser‐Greenhouse, Ṧidak's comparison, Alpha = 0.05, 95% CI. G) Enucleation% of the terminally differentiated MC‐HeO‐derived HSPCs to RBCs, based on flow cytometry using CD235/CD71 and DRAQ5 staining, *n *≥ 6, unpaired Mann–Whitney test, 95% CI.

Phase II of the original system (Figure 2A) utilizes adherent surfaces and static culturing to form proficient HeOs. However, their translation to a scalable system requires a dual transition to both suspension and dynamic culturing. Applying dynamic conditions to an adherent surface did not compromise the HeO‐forming potential (**Figure** **3**A); despite the turbulence, adherence to the tissue culture (TC) surface did occur (Figure S3A, Supporting Information). Similarly, when a non‐adherent surface was provided in a static environment, HeO formation remained unaffected (Figure 3A). In contrast, when both conditions were combined (non‐adherent surface and dynamic culturing), HeO formation, as well as weekly HSPC production per EB, were negatively affected (Figure 3A,B), resulting in an average 4.5‐fold decrease in the total HSPC/EB yield compared to the control (Figure 3B). These observations highlighted that while non‐adherent surfaces and dynamic culturing individually supported HeO formation, their combination failed to achieve the HSPC yields needed for clinically relevant iRBC production. Consequently, this combined approach was not selected, underscoring the need for a more effective strategy. To address this, we investigated microcarriers (MCs) as adherent surfaces, providing sufficient area for HeO formation in turbulent culture systems. Three different MCs were tested: Cytodex 1 (Cdex1); Cytopore 1 (CP1); Cytopore 2 (CP2). All tested carriers are positively charged and GMP‐compatible. Cdex1 is non‐porous, while CP1 and CP2 are microporous with varying charge densities. EBs were generated through the optimized single cell/dynamic platform and cultured together with 2 g L^−1^ MCs in a LA culture vessel with continuous mixing at 55 rpm. The MC testing was carried out on a small scale, using LA 24‐well plates seeded with 5 EBs per well. Upon addition of MCs, an immediate attraction was observed, with single EBs attracting multiple MCs (Figure 3C). In the first week, the EBs started to form HeOs while embedding more MCs over the subsequent weeks (Figure 3C). The HeO‐forming potential per EB was found to be similar between the MCs and was not significantly different from the control situation of non‐MC tissue culture, confirming that HeOs can indeed be formed on MC surfaces within a dynamic environment (Figure 3D). Production of HSPCs was observed starting from the fourth week for all three MCs. Both hematopoietic (CD34, CD45, CD43) and erythroid (CD235) content within the produced HSPCs exhibited slight variations at week five across all MCs, with the patterns becoming comparable to the TC control in the subsequent weeks. The most pronounced differences in expression patterns of HSPCs were observed with CP1 over the weeks (Figure 3E). The CD34^–^/CD45^+^ cells, representing progeny already committed to nonerythroid lineages, were slightly more abundant in all MCs‐derived HSPCs compared to the TC control (Figure 3E). However, further erythroid differentiation of HSPCs originating from MC‐HeOs resulted in a population that was >90% pure erythroid across all three MCs (Figure S3B). Thus, the presence of non‐erythroid cells prior to terminal erythroid differentiation posed a negligible risk in the final RBC product. The average fold expansion of the produced HSPCs in erythroid expansion/differentiation media was similar between TC control and Cdex1, while significantly lower when CP1 or CP2 were used (Figure 3F). The enucleation rate remained unaffected by the use of MCs and stayed within the 46–71% range, similar to the TC control, as quantified by flow cytometry (Figure 3G). Although all three MCs were supporting HeO formation, Cdex1 was selected due to its ability to achieve the highest HSPC expansion rate.

The results presented here demonstrate that Phase II of the protocol can be carried out in a dynamic environment. By using MCs—which provide a surface for EB adherence absent in larger culture vessels (e.g., erlenmeyers or bioreactors)—efficiency can reach levels comparable to the control non‐MC tissue culture condition.

### 2.4 Erythroid Expansion and Terminal Differentiation can Occur Under Dynamic Culture Condition

**Figure 4:**
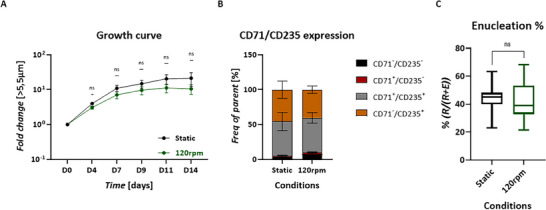
Erythroid expansion and maturation can occur under dynamic culture condition. A) Expansion potential of differentiating HSPCs under 120 rpm shake‐speed. The quantifications are shown with mean and SEM, *n *≥ 10, Mixed‐effect with Geisser‐Greenhouse, Ṧidak's comparison, Alpha = 0.05, 95% CI. B) Erythroid marker expression pattern of terminally differentiated HSPCs using 120 rpm agitation, *n *≥ 6, Mixed‐effect with Geisser‐Greenhouse, Ṧidak's comparison, Alpha = 0.05, 95% CI. C) Enucleation% of the iRBCs, based on flow cytometry using CD235/CD71 and DRAQ5 staining, *n *≥ 9, unpaired Mann–Whitney test, 95% CI.

To transition the entire platform to dynamic culturing, Phase III was also investigated for its applicability in turbulent environments. The harvested HSPCs were expanded and terminally differentiated in a static (CTRL) or a dynamic environment using an orbital shaker. The static CTRL samples were cultured on TC dishes, while the dynamic samples were cultured in low‐attachment surfaces using LA‐plates and Erlenmeyer (ELM) flasks. Adult erythroblast (EBLs) have previously demonstrated resilience to turbulent environments.^[^ ^28^, ^29^, ^30^ ^]^ Here, three shaking speeds were tested on one cell line: 55 rpm (as used in Phase I‐II), 120 and 250 rpm. The highest growth was observed at 250 rpm, although it was still lower than the static control (Figure S4A, Supporting Information), while enucleation was hampered at 250 rpm, resulting in the lowest reticulocyte (RET) survival (Figure S4B, Supporting Information). Based on the initial testing, 120 rpm was selected for further examination using multiple cell lines. The average fold change was not significantly different between 120 rpm or static conditions (**Figure** **4**A). Moreover, at the end of terminal differentiation (day 14), no significant differences were observed between the conditions in terms of their erythroid marker expression (CD71/CD235) pattern (Figure 4B) and enucleation rate (Figure 4C).

In conclusion, Phase III of the protocol can be executed in dynamic culturing using 120 rpm on an orbital shaker, demonstrating its feasibility for translation to larger‐scale dynamic systems.

### 2.5 The Complete iRBC Differentiation Platform Implemented in a Scalable Dynamic Environment

**Figure 5:**
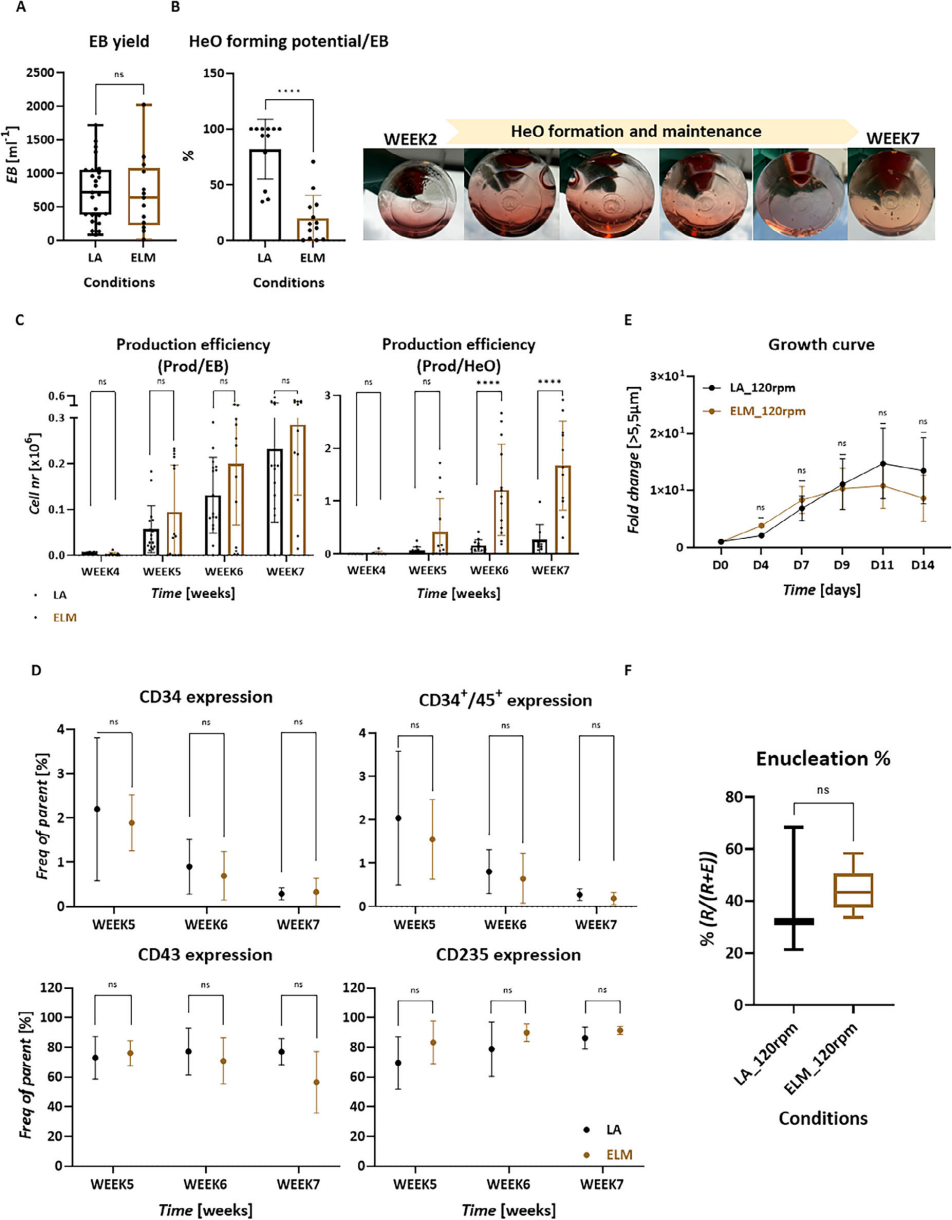
The entire 3D‐iRBC differentiation platform carried out in dynamic environment using MCs and ELM on shaker. A) Comparison of EB yield using Low attachment plates versus ELMs, *n *≥ 13, unpaired Mann‐Whitney test, 95% CI. B) Quantification of HeO forming potential (left), *n *≥ 12, unpaired Mann–Whitney test, 95% CI, and HeO morphology grown on Cdex1 in ELM and shake platform (right). C) Weekly production efficiency of HSPCs derived from MC‐HeOs. D) Weekly marker expression pattern of HSPCs derived from MC‐HeOs using flow cytometry, *n *≥ 9, unpaired Mann‐Whitney test, Holm–Šídák method, Alpha = 0.05. E) Expansion potential of differentiating HSPCs, that cultured in shake platform. The quantifications are shown with mean and SEM *n *≥ 4, Mixed‐effect with Geisser–Greenhouse, Ṧidak's comparison, Alpha = 0.05, 95% CI. F) Enucleation% of the iRBCs derived in agitation culture, based on flow cytometry using CD235/CD71 and DRAQ5 staining, *n *≥ 3, unpaired Mann‐Whitney test, 95% CI.

To further assess scalability, we performed the entire platform in ELMs on orbital shaker (Figure 2A, optimized platform). The 125 mL ELMs were inoculated with 0.3–0.6 × 10^6^/mL iPSCs using the optimized single cell/dynamic method (Ph.I). The average number of formed EBs was similar between ELM and LA‐plates (**Figure** **5**A). These EBs were then co‐cultured with Cdex1 MCs in ELMs under continuous mixing. HeO formation was observed from week 1, and their growth was visible over the subsequent weeks (Figure 5B). The HeO forming potential was significantly lower when ELM was used contrast to LA 24‐ or 6‐well plates (Figure 5B). While the weekly HSPC yields per EBs were similar between the two types of culture vessel (Figure 5C). However, the weekly HSPC yields per HeOs were significantly higher in ELM contrast to LA‐ plates, resulting in 6.5 times higher overall HSPC production (between week 4–7), suggesting the derivation of more potent organoids in ELMs (Figure 5C). Culturing on MC surface in ELMs did not result in alterations in the expression pattern of markers on the produced HSPCs, as quantified by flow cytometry (Figure 5D). The average fold expansion of the HSPCs did not differ significantly between LA and ELM using 120 rpm (Figure 5E). The enucleation rate was not impacted by the use of ELM in combination with MCs and remained similar to controls, as quantified by flow cytometry (Figure 5F).

We thus demonstrated that differentiation in dynamic culturing can be scaled up successfully by performing the entire protocol from iPSC to enucleated iRBCs in ELMs using orbital shakers.

### 2.6 In Vitro Assays Confirming Functional, Definitive Erythroid Characteristics

**Figure 6:**
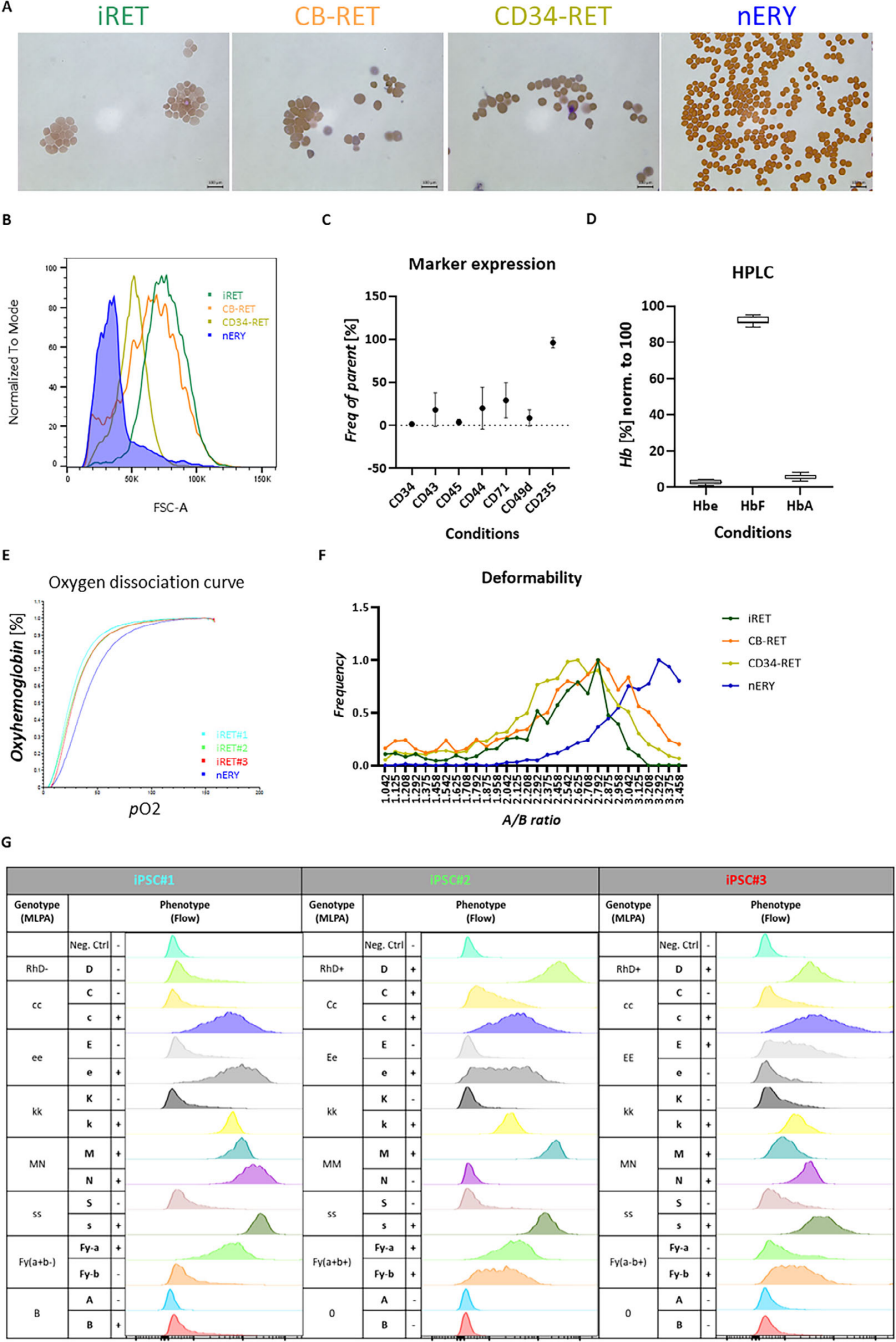
In vitro assays confirming functional, definitive erythroid characteristics of the terminally differentiated iRBCs. A) Cytospins combined with benzidine‐giemsa staining on filter‐purified RETs and on nERYs. B) RET‐size measurement by flow cytometry on CD235^+^/DRAQ5^‐^ cell population. C) Marker expression pattern of the iRBCs using flow cytometry, *n *≥ 58. D) Quantification of Hbe (α2ε2), HbF (α2γ2) and HbA (α2β2) based on HPLC tracks performed on iRBCs, *n *= 8. E) Oxygen dissociation curve of iRBC lines measured by the Hemox analyzer. F) Deformability assay or RETs and nERY measured by ARCA. G) Blood group phenotype measured on iPSC lines by DNA‐based (MLPA and SNP array)‐ and on iRBCs by protein‐based (flow cytometry)‐ assays, *n *≥ 3.

Next, the functionality of the generated iRBCs with our platform was assessed. Wherever applicable, native erythrocytes (nERYs) and/or CB/PBMC/CD34‐derived cRBCs were used as controls. Purified, enucleated iRBCs were hemoglobinized and exhibited morphological similarities to cord‐ and adult counterparts, as demonstrated by cytospin (**Figure** **6**A). Consistent with previous findings, the iPSC‐derived reticulocytes (iRETs) were larger in size than cultured CD34‐derived RETs, but similar to CB‐RETs as quantified by flow cytometry (Figure 6B). The hematopoietic‐ and erythroid‐marker expression pattern of the end‐stage culture was measured by flow cytometry, showing 96% erythroid purity marked by CD235 positivity (Figure 6C). The enucleation rate ranged between 48% and 62%, measured by DRAQ5 staining in flow cytometry (Figures 2G, 3G, 4C, 5F). iRBCs expressed HbF with near absence of Hbe and low expression of HbA, as shown by HPLC (Figure 6D). The oxygen affinity of iRBCs was measured by hemox analyzer. Due to high HbF content, iRBCs exhibited higher oxygen affinity than native HbA‐expressing ERYs (Figure 6E). The deformability potential of iRET was measured by automated rheoscope and cell analyzer (ARCA) and was lower compared to nERYs, but was comparable to reticulocytes derived from adult‐ or CB‐CD34^+^ cells (Figure 6F). The blood group phenotype of the iRBCs was measured by protein‐based flow cytometry assay, which exhibited the same patterns as the genotyping (DNA‐based assay) performed on the iPSC lines of origin (Figure 6G).

Thus, in vitro molecular and cellular assays confirmed that the iRBCs derived by our differentiation platform are functional and possess a fetal phenotype, consistent with earlier reports.^[^ ^31^ ^]^

### 2.7 In Vivo Maturation and Clearance of iRBCs in a Mouse Model is Comparable to Native RBCs

**Figure 7:**
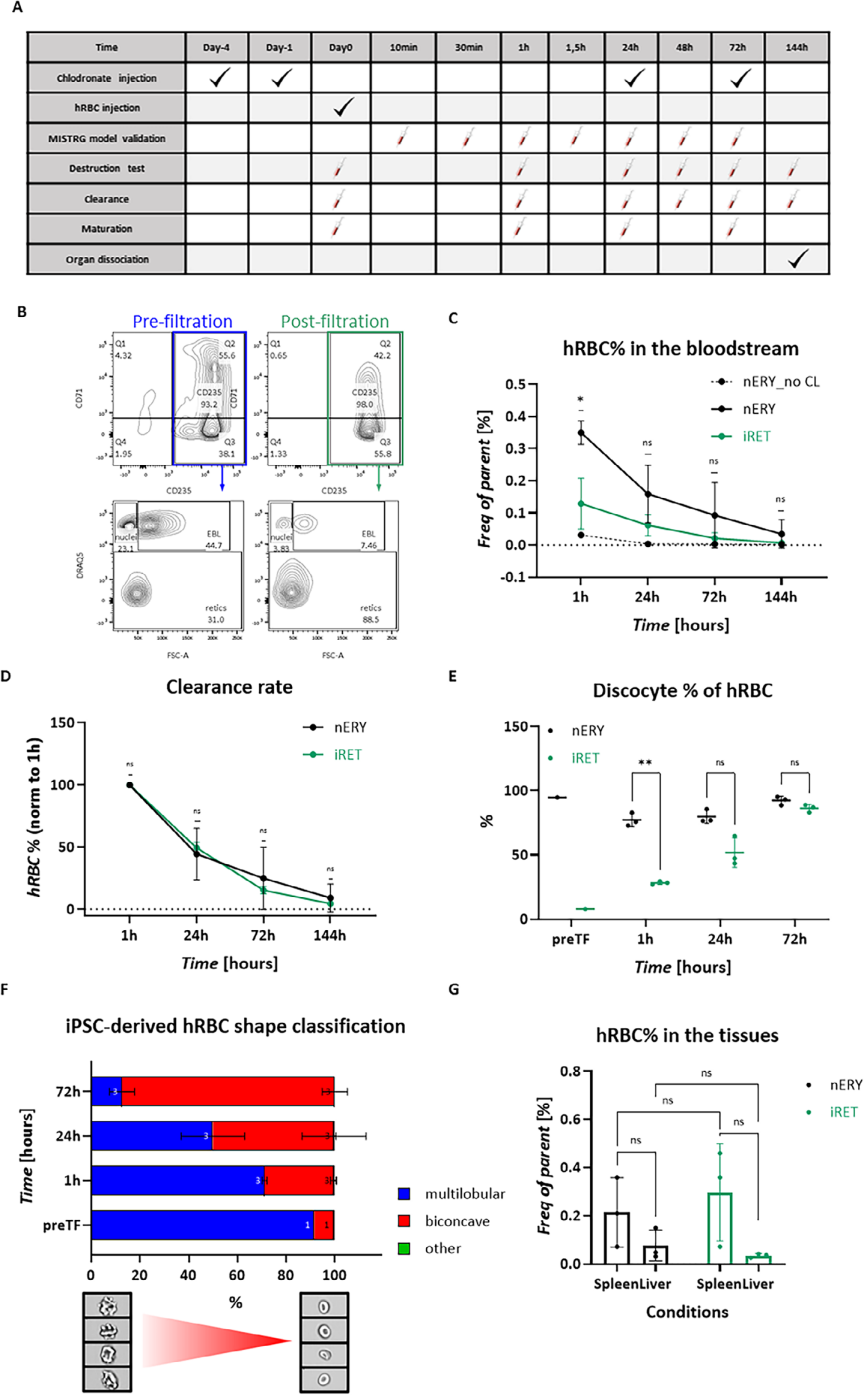
In vivo maturation and survival of iRETs is shown in MISTRG mouse model. A) Timeline of transfusion assay. B) Filtration of iRETs shown by flow cytometry using CD235/CD71 and DRAQ5 staining. C) Quantification of hRBC% in the mouse blood using flow cytometry, *n *= 3, Mixed‐effect with Geisser‐Greenhouse, Tukey's comparison, Alpha = 0.05, 95% CI. D) Clearance rate of hRBC from the mouse bloodstream using flow cytometry, *n *= 3, two‐way ANOVA with Geisser‐Greenhouse, Ṧidak's comparison, Alpha = 0.05, 95% CI. E) The of biconcave human erythrocytes after transfusion, quantified by ImageStream, *n *= 3, Mixed‐effect with Geisser‐Greenhouse, Ṧidak's comparison, Alpha = 0.05, 95% CI. F) Morphological changes of the injected iRBCs in the bloodstream along time quantified by ImageStream, *n *= 3. G) Distribution of hRBCs in the mouse spleen and liver, *n *= 3, nERY versus iRET: unpaired Mann‐Whitney test, Holm‐Šídák method, Alpha = 0.05; Spleen versus Liver: two‐way ANOVA, Ṧidak's comparison, Alpha = 0.05, 95% CI.

The circulation of human RBCs (hRBCs) was investigated in the immunodeficient MISTRG mouse model to assess their in vivo survival and maturation.^[^ ^32^ ^]^ To validate the mouse model in the transfusion assay, nERYs were initially injected. Phagocyte depletion was established by liposome‐chlodronate injections, administered 4 and 1 day prior to transfusion, to reduce clearance of hRBCs. Subsequently, 20 × 10^6^ hRBCs (0.1% hRBC of total murine blood) were injected into the tail veins of the mice (for timeline see **Figure** **7**A). 10 mins post‐transfusion 0.08% hRBCs were measured by flow cytometry, which slightly dropped at 30 min and remained constant in the following hour (0.062–0.069%) representing the initial destruction plateau (Figure S5A, Supporting Information). Subsequent timepoints showed a gradual clearance of hRBCs (Figure S5A, Supporting Information). 72h post‐transfusion, the spleen, liver, lung, and bone marrow were dissociated and analyzed for their hRBC content using flow cytometry. As expected, the highest number of cells resided in the spleen, suggesting that the majority of clearance took place there (Figure S5B, Supporting Information).

Next, iRETs were injected into MISTRG mice to assess their maturation pattern from RETs to erythrocytes and their short‐term survival and clearance in vivo (Figure 7A). The 180 × 10^6^ hRBCs (1% hRBC of total mouse blood) were injected into the tail veins of each mice. Based on the initial destruction determination (Figure S5A), the first blood draw timepoint was 1 hour post‐transfusion. Prior to transfusion, the iRETs were filtered using leukocyte depletion filter. The iRET purity after filtration was 88.5% (CD235^+^/DRAQ5^‐^) of which 39.9% was CD71^+^ indicating immature RETs, as quantified by flow cytometry (Figure 7B). The average hRBC% 1 hour post‐transfusion was significantly higher in nERYs (0.35%) than in iRETs (0.13%) and was minimal in non‐clodronate mice (0.031%), highlighting the necessity of phagocyte depletion in this murine model (Figure 7C). In chlodronate‐depleted mice, both the nERYs and iRETs gradually disappeared and were cleared entirely by 144 h (Figure 7C). Human RBC percentage was normalized to 100% at 1 h post‐transfusion to compare the rate of clearance between control and sample groups. There was no significant difference between the clearance rate of nERYs and iRETs (Figure 7D). Furthermore, at 1 h post‐transfusion of iRETs the CD71 positivity decreased to an av. of 14.63% which further decreased to av. The 7.33% at 24 h indicating rapid reticulocyte maturation (Figure S5C). Besides CD71, the morphological characteristics of the injected cells served as an indicator for assessing the maturation pattern of iRETs in the murine bloodstream, which was investigated by ImageStream (Figure S5D, Supporting Information). This data, combined with Amnis AI analysis, allowed for the classification of cell shapes into three distinct categories: I) multilobular, II) biconcave discocytes, and III) other forms. Native erythrocytes were used as control, with 94.77% of the cells exhibiting a biconcave discocytes shape prior to injection, a proportion that remained stable over the 72 h post‐transfusion (Figure 7E). The iPSC‐derived cells before injection displayed only 7.91% biconcave discocytes, but this figure increased to an av. of 86.4% at 72 h post‐transfusion, aligning closely with the nERY control (Figure 7E; Figure S5D, Supporting Information). The proportional changes of the three categories in the iPSC‐derived cells were quantified over time, revealed a predominance of either multilobular I) or biconcave II) cell shapes. Prior to transfusion, an av. of 91.4% of the iPSC‐derived RBCs were multilobular, indicative of a predominantly high reticulocyte presence (Figure 7F). At 1 h post‐transfusion, a quarter of the cells underwent morphological transformation to become biconcave discocytes (Figure 7F). This transformation increased to 50% at 24‐h post‐transfusion, and by 72 h, nearly the entire cell population was biconcave, with only 12.6% retaining a multilobular shape (Figure 7F; Figure S5D, Supporting Information). The 144 hours post‐transfusion, the spleen and liver were dissociated and analyzed for hRBC content using flow cytometry. Both nERY and iRET distributions were higher in the spleen than in the liver, although this difference was not significant. There was no significant difference between the percentage of nERY and iRET in the different tissues (Figure 7G).

## 3 Discussion

In this study, we present several key advances in iPSC‐to‐RBC differentiation that contribute to a robust, scalable platform through the transition from traditional static/adherent culture to dynamic suspension‐based conditions. The workflow was streamlined by directly converting 3D‐iPSC aggregates to EBs, incorporating a brief 3D‐iPSC phase that opens the possibility of omitting 2D‐iPSC maintenance entirely in future optimizations. Derivation of 3D‐iPSCs from single cells significantly reduced enzymatic treatment time, enabling a rapid, scalable, and high‐throughput process. Most notably, this approach enabled controlled and homogeneous EB formation, leading to markedly enhanced organoid formation potential and improved reproducibility, addressing a prior critical limitation. Integration of pre‐formed EBs with MCs provided a surface for organoid formation in suspension, creating a versatile platform adaptable for generating various organoid types beyond hematopoietic lineages. Notably, we demonstrated high‐efficiency iPSC‐to‐RBC differentiation with robust enucleation, achieved entirely in a dynamic, suspension‐based culture system, a rare accomplishment not previously demonstrated under dynamic conditions. This system is fully compatible with dynamic, scalable bioprocessing approaches, ensuring homogeneous media mixing and potential for GMP‐compliant large‐scale production, enabled by an entirely suspension‐based workflow that eliminates static culture steps and facilitates automation and closed‐system integration.

With the optimizations and conversion to dynamic culturing in Phase I, the reproducibility and efficiency of the system were significantly enhanced, including more consistent HeO formation, alleviating earlier limitations. Furthermore, we have demonstrated that MCs can replace traditional culture surfaces during Phase II and promote RBC‐proficient HeO formation in a turbulent environment. Interestingly, HeO formation and HSPC production efficiency were influenced more by vessel format than by surface type. MCs in Low‐attachment plates promoted better HeO formation but resulted in lower HSPC yields compared to ELM flasks. This may be related to variations in fluid dynamics between the two culture vessels, and further research needs to be performed to understand the basis of these differences.^[^ ^33^ ^]^ The third phase of the platform showed comparable expansion rates between static and dynamic conditions, indicating that iPSC‐ERY progenitor cells and their differentiated counterparts tolerate the shear stress generated in shake flasks better than initially expected. Although a slight decrease in yield was observed in shake flasks, this difference was not statistically significant. It remains possible that shear stress contributes to subtle changes in signaling or molecular pathways, as described in previous studies, which might not be fully reflected in overall yield but could affect other aspects of cell behavior.^[^ ^29^, ^30^ ^]^ This slight reduction in yield under dynamic conditions is expected to be compensated in bioreactors, similar to the results we observed in adult RBC differentiation, given the lower overall shear stress in stirred‐based reactors, but these effects need to be further studied.^[^ ^28^ ^]^ Based on the measured values, it was determined that a minitransfusion unit required for Phase I clinical trial (1 × 10^11^ enucleated iRBCs) can be generated from 4.9 × 10^7^ iPSCs. This estimation is derived from dynamic culturing results using ELM flasks and MCs (Figure 2A, platform 2.), which are currently used as a transitional step toward implementing bioreactors. Stirred tank bioreactors are anticipated to significantly reduce variation in HPSC production efficiency and enhance fold expansion due to their highly controllable parameters, such as oxygenation, pH, and mixing speed, which we are currently pursuing.

It is noteworthy that organoid formation was achievable without the use of MCs or adherent surface in a dynamic environment using our optimized protocol, though with a threefold reduction in total HSPC production efficiency (Figure 3B vs. Figure 5C). The use of MCs in this context highlights their utility in enabling efficient large‐scale production by providing surface area and presumably offering protection of the organoids against hydrodynamic stress. We propose that adherence, by means of TC surface or MCs acts as a crucial selection process, favoring EBs with successful/more potent HeO formation and thereby enhancing HSPC yield. We speculate that adherence may allow molecular signaling and cellular interactions essential for forming organized tissue‐like structures typical of organoids.^[^ ^34^, ^35^, ^36^ ^]^ This finding aligns with observations from other organoid‐based systems, where surface adherence is a critical factor.^[^ ^37^, ^38^, ^39^ ^]^ Continued exploration into the molecular mechanisms underlying the dependence on adherence will undoubtedly provide further insights into optimizing HeO cultivation for broader scientific and clinical benefits. However, we also recognize that the use of MCs may introduce additional considerations, such as system complexity and the need for enhanced safety measures prior to human administration. Their eventual application will require a careful risk‐benefit analysis.

During the optimization process, iPSCs were initially maintained on matrix‐requiring 2D culture systems, before being converted into 3D‐iPSC aggregates (+bFGF) two to five days prior to EB formation (‐bFGF). Extension of the 3D‐iPSC maintenance would allow for omitting the 2D‐iPSC maintenance step, further streamlining the process. 3D‐iPSC maintenance in a shake flask or bioreactor is readily available by various companies, allowing large‐scale applications. To further facilitate large‐scale culturing, we have identified two strategically relevant cryopreservation timepoints that enable more efficient planning. During Phase I, single‐cell treated and prefiltered iPSCs can be cryopreserved in large quantities and used to directly inoculate culture vessels. During Phase II, HPSC production cells are continuously produced and harvested over subsequent weeks. These cells can be cryopreserved and later cultured without yield loss compared to fresh cells (data not shown). In addition, we observed >90% erythroid purity and similar enucleation rate, regardless of the harvest week, indicating that cryopreserved HSPC production cells can be combined upon thawing.

In agreement with previous findings, the derived iRBCs resemble a fetal phenotype, exhibiting higher O_2_ affinity than adult‐RBCs. The existence of a naturally occurring mutation in a healthy human cohort living with elevated HbF or hereditary persistence of Hb underscores the biological compatibility of HbF‐RBCs in normal physiological conditions.^[^ ^18^ ^]^ Moreover, partial expression of HbF is considered a viable therapeutic approach for patients with sickle cell disease and β‐thalassemia.^[^ ^19^, ^40^, ^41^, ^42^ ^]^ Previous demonstrations of the safe use of HbF high CB‐ERYs in children and adult recipients further support the potential applicability of iRBCs in transfusion medicine.^[^ ^17^, ^43^ ^]^ Based on these lines of evidence, we suggest that the use of HbF‐expressing iRBCs in adults is a compelling therapeutic option. This product may even be more relevant, particularly for pre‐term infants, where current transfusions using HbA‐containing blood correlate with adverse events.^[^ ^20^, ^44^, ^45^, ^46^, ^47^ ^]^ However, regulatory approval for pediatric use is typically more stringent than for healthy adults.

iRETs were injected into highly immunodeficient MISTRG mice to assess their clearance kinetics and maturation timeline toward erythrocytes, for the first time. The immediate clearance was notably higher for iRETs than native erythrocytes at 1 h post‐transfusion, possibly due to inherent cellular differences between reticulocytes and mature erythrocytes. Additionally, it is also possible that the effect of in vitro culturing contributed to this difference. Importantly, the clearance rate of iRBCs became comparable to that of native erythrocytes after this initial period (1 h). Notably, human erythrocytes, native or in vitro‐derived, exhibit accelerated clearance rates across diverse murine models, even under conditions of phagocyte depletion.^[^ ^10^, ^48^, ^49^ ^]^ Under normal physiological conditions, human erythrocytes demonstrate a lifespan of ≈120 days. The discrepancy between the typical lifespan of human erythrocytes and their accelerated clearance in murine models highlights the necessity for alternative methodologies to accurately assess the in vivo survival kinetics of hRBCs. However, mouse models remain valuable for investigating the transition of immature RETs to mature ERYs, as we demonstrated that iRETs were able to transition from a multilobular to a biconcave shape in the murine blood stream, indicative of reticulocyte to erythrocyte maturation. This maturation process followed the classical erythroid maturation pattern, with visible changes as early as 1 h postinjection and by 72 h nearly all detected human cells exhibited erythrocyte morphology. However, due to the ongoing clearance dynamics it remains challenging to definitively determine whether the initially injected erythrocytes persist at 72 h while the multilobular cells were cleared or new maturation events have been occurred. Future studies will be essential to track cell fate accurately.

A current limitation on the production of iPSC‐derived blood in vitro is the cost of commercially available media used during the organoid formation phase, rendering it unaffordable for large‐scale bioreactor culturing. To overcome this, we are currently evaluating in‐house media formulations and exploring strategies to achieve economically viable solutions.^[^ ^5^, ^50^, ^51^ ^]^ Additional technical challenges to be solved prior to clinical application include the development of an efficient method for purifying reticulocytes. Current practice in the field applies leukocyte filters, which demonstrate suboptimal filtration efficiency (≈30%), leading to substantial cell loss during the final production stage. As no alternative options are currently known, innovative solutions are necessary in this area.

## 4 Conclusion

In conclusion, the 3D‐iRBC differentiation platform described above yields erythroid cells that are inherently fetal in nature and capable of enucleation at a high rate, even in a dynamic environment, representing a significant advancement in the field. RBCs derived from iPSCs using this platform have demonstrated functionality through in vitro molecular and cellular assays. Moreover, in vivo survival and maturation assays in mice—conducted for the first time with purified iPSC‐derived reticulocytes—further validated the functionality and potential of these RBCs as transfusion products. Our platform is scalable and GMP‐compatible, meeting clinical standards and offering significant promise for transfusion medicine. Additionally, it establishes a crucial link to bioreactor applications, facilitating the potential for large‐scale implementation.

## 5 Experimental Section

### Study Design

Five iPSC lines were used in this study from healthy donors. In each experiment, either at least three technical replicates (from the same cell line) or three biological replicates (from different cell lines) were included, depending on the type of experiment. Technical replicates were applied for assay reproducibility, while biological replicates were used to account for variability among cell lines. Sample sizes are provided in the figure captions and/or visualized in the figures. All data were included unless technical issues were identified (e.g., handling errors, equipment malfunctions). Outliers were not excluded and are represented in scatterplots and boxplots. The experimental units were cell cultures, while mice were considered independent biological samples for in vivo experiments. Mice were randomly selected from the same treatment group. The treatment administration was performed by a caretaker who was aware of group assignments. Mouse‐ blood and tissue sample collection and analysis were conducted by an investigator who was not blinded to the treatment groups. The study was conducted as a controlled laboratory experiment, with the specific quantification methods detailed in the Materials and Methods section.

### Experimental Details—Chemicals

The chemicals were purchased from Merck (Germany) or Sigma‐Aldrich (Germany), the culture reagents from Thermo Fisher Scientific (USA) or from Stem Cell Technologies (Canada), unless otherwise specified.

### iPSC Lines

Informed consent was given in accordance with the Declaration of Helsinki and the Dutch national and Sanquin internal ethic boards.

Five iPSC lines were used in this study, which were generated by us from healthy donor materials. MML‐6838‐Cl2;^[^ ^52^ ^]^ SANi003‐A;^[^ ^53^ ^]^ BEE6000.cl3: PBMC‐derived erythroblast origin, episomal plasmid; MES12832.cl2: MPB‐derived erythroblast origin, sendai‐virus vectors, CES11127.cl4: CB‐derived erythroblast origin, sendai‐virus vectors.

### Cell Culture

All cells were cultured at 37 °C in a humidified atmosphere containing 5% CO_2_.

### iPSC Maintenance

The iPSCs were cultured on Matrigel (BD Biosciences) or on Geltrex (used interchangeably) in essential 8 medium (E8) during weekdays and in mTESR+ on weekends. Passaging occurred every 5–7 days using ReLeSR, E8 + CloneR supplement according to the manufacturer's instructions.

### 2D Erythroid Differentiation of iPSC

The 2D‐iRBC differentiation protocol is detailed in Hansen et al.^[^ ^27^ ^]^

### 3D Erythroid Differentiation of iPSC

The original 3D‐iRBC differentiation system was developed and detailed by Bernecker et al.^[^ ^24^ ^]^ Briefly, adherent iPSC‐colonies were detached with collagenase IV and seeded on LA‐plates. EBs were cultured for 5 days in hESC medium without bFGF. The 10–15 EBs were then transferred to 6‐well plates with APEL2 medium supplemented with PFHM‐II, IL3, SCF, and EPO. Medium was changed weekly. HSPCs released into the supernatant were cultured for 18 days in Iscove's medium with human plasma, insulin, and holotransferrin, stimulated with SCF, IL3, and EPO following a three‐step erythroid differentiation protocol.

A stepwise translation to agitation culturing performed by us resulted in an optimized 3D‐iRBC differentiation platform containing three phases. All three phases were carried out on an orbital shaker (Thermo Fisher Scientific, #88881 102) applying 55 rpm during Phase I‐II and 120 rpm at Phase III.

*Phase I) 3D‐iPSC and EB Formation*: 2D‐iPSC colonies were treated with Accumax, then passed through on a strainer (40 µm) to obtain single‐cell suspension. A cell concentration of 0.3–0.6 × 10^6^ iPSC/ml was used to inoculate 6‐well LA‐plates or 125 mL ELMs in hESC media containing DMEM/F12 (Thermo Fisher Scientific #21331046l), L‐glutamine (2 mm), 1X Pen/Strep (100X stock), Knock out serum replacement (20%), 1X Non‐essential amino acids (100X stock), β‐mercaptoethanol (100 µm), supplemented with thermostable bFGF (10 ng mL^−1^) and Rock inhibitor (10 µm). Two to five days post‐inoculation the media were refreshed with hESC media without growth factors to initiate EB formation, and the aggregates were cultured for an additional 5 days. EB morphology was monitored by microscopy, and the number of EBs was quantified before downstream application.

*Phase II) HeO Formation: The* 2–4 EBs mL^−1^ in 24‐well LA, 6‐well LA or 10–17 EBs mL^−1^ in 125 mL ELMs were seeded together with MCs (2 g L^−1^; Cytiva, #17044899; #17091199; #17127199) using APEL2 media supplemented with PFHM‐II Protein‐Free Hybridoma Medium (5%), L‐glutamine (2 mm), 1X Pen/Strep (100X stock), IL3 (5 ng mL^−1^), SCF (100 ng mL^−1^) and EPO (3 U mL^−1^). One week later, the media was doubled without removal, followed by weekly media refreshments. Four days after each media refreshment, growth factors were added to the cultures. The produced HSPCs were continuously harvested between weeks 4–7 at each media refreshment and either stored frozen or differentiated into enucleated RBCs in separate suspension cultures.

*Phase III) ERY Differentiation*: the differentiation was carried out for 14–18 days. HSPCs were seeded either onto TC plates, serving as static controls, or onto LA‐plates and ELMs for dynamic cultures. The seeding density was 0.8 × 10^6^ mL and maintained at 0.5–1 × 10^6^ mL with bi‐daily half‐media volume refreshments. From day 11 onward, a concentration of 2 × 10^6^/mL was used without further media change. ErythroPlus (PAN biotech) base media was applied, supplemented with holotransferrin (1 mg mL), HSA (0.1%), L‐glutamine (2 mm), 1X Pen/Strep (100X stock), Omniplasma (10%, Octopharma, Wien, Austria), heparin (5 U mL), EPO (3 U/ml). Day 0–7 IL3 (5 ng mL) and day 0–11 SCF (100 ng mL) were also added in the media.

### Primary Cell Culture

Informed consent was given in accordance with the Declaration of Helsinki and the Dutch national and Sanquin internal ethic boards.

MPB‐derived EBLs and CB‐derived EBLs were cultured as described previously^[^ ^5^ ^]^ Briefly, mononuclear cells were cultured in expansion medium ErythroPlus (PAN biotech) containing holotransferrin (0.3 mg mL), HSA (0.1%), L‐glutamine (2 mm), 1X Pen/Strep (100X stock), EPO (2 U/ml), SCF (100 ng mL^−1^), dexamethasone (1 µm). IL3 (1 ng mL^−1^) was added on the first day of culture. Terminal differentiation was induced by EPO (10 U mL^−1^), Omniplasma (5%, Octopharma, Wien, Austria), holotransferrin (1 mg mL^−1^) and heparin (5 U mL^−1^).

### HPLC

Culture lysates were prepared and stored at −80 °C before analysis as described previously.^[^ ^54^ ^]^ In short, Hb separation was performed by high‐performance cation exchange liquid chromatography (HPLC) on Ultimate 3000 equipment (Thermo Fisher Scientific) using 30 min of elution over a combined NaCl (20–200 mm) and pH 7.0 to 6.6 gradient in BisTris/HCl (20 mm) and KCN (2 mm). A PolyCAT A 100/4.6 mm, 3 mm, 1500 Å column (PolyLC, Columbia, MD) was used.

### Cytospins and Staining

Cells were cytospun (Shandon CytoSpin II Cytocentrifuge) onto 76 × 26 mm glass microscope slides. Cells were stained with conventional benzidine‐giemsa reaction, labelling hemoglobin (brown) and nuclei (purple). In short, cells were fixed in methanol, stained with benzidine (O‐diasidine (1%)) followed by treatment with H2O2 (1%) solution. Giemsa staining was carried out using Differential Quik III Stain Kit (Polysciences #26 419). All slides were rinsed in deionized water, air‐dried, and analyzed with Leica DM2500 microscope.

### Flow Cytometry

Marker expression was measured on an LSR‐II (BD Bioscience) and analyzed using Flowjo software (Flowjo, Ashland, USA). The used antibodies are listed in Table S2 (Supporting Infrmation).

### Microscopy and EB Characterization

Morphological images were acquired using an EVOS XL microscope (Thermo Fisher Scientific) with a 4× objective (AMEP‐4632). Five‐day‐old EBs were individually plated into wells of a 24‐well plate and imaged. EB parameters, including diameter and shape, were measured using ImageJ software (version 1.53e). Roundness and irregularity were quantified in ImageJ and additionally evaluated by visual inspection. EB size was determined by measuring the area (µm^2^) of each individually imaged EB in ImageJ. Each EB was measured three times, and the mean area was calculated. Measurements with a standard deviation exceeding 2000 µm^2^ were excluded. Plated EBs were individually differentiated according to phase II of the protocol. HeO formation potential was assessed microscopically and correlated with EB size. To evaluate EB size in relation to erythroid proficiency, HSPC potential from each HeO was recorded and correlated with the corresponding EB size.

### Oxygen Dissociation

The Oxygen dissociation curve (ODC) of RBCs was measured by Hemox Analyzer (TCS scientific Corp.). RBCs were re‐suspended in PBS + HSA (0.5%; human serum albumin). The measurements were performed in PBS (5 mL) + HSA (0.5%) + Antifoam Y‐30 emulsion (50 µL; 1%; Sigma Aldrich, #A5758). The ODC is recorded during deoxygenation with nitrogen gas and plotted on graph paper; the oxygen tension is detected by a Clarke electrode while the oxyhemoglobin fraction (%HbO_2_) is evaluated by a dual‐wavelength spectrophotometer. The ODC is obtained during deoxygenation with nitrogen gas and plotted. Oxygen tension is measured with a Clarke electrode, and the oxyhemoglobin fraction (%HbO_2_) is determined using a dual‐wavelength spectrophotometer. The OEC software was used for data collection and analysis.

### Deformability

The deformability of RBCs was evaluated using an Automated Rheoscope and Cell Analyzer (ARCA; ARCA‐Linkam CSS450), where cells were elongated by shear flow in a viscous medium (Polyvinylpyrrolidone) between parallel glass plates.^[^ ^55^ ^]^ Automated imaging algorithms identified cells and calculated their deformability. Experiments were performed at 10 Pa shear stress. The elongation extent (major radius/minor radius) was plotted against the normalized frequency of occurrence. In vitro‐derived RBCs were filtered prior to measurement to purify RETs using Acrodisc WBC filter. For the measurement and analysis ARCA program was used.

### Blood Group Phenotyping

The multiplex ligation‐dependent probe amplification (MLPA) and SNP array were carried out by the Sanquin Diagnostic Department (Amsterdam, The Netherlands). The protein‐based phenotyping was completed by conventional immunostaining coupled with flow cytometry (Canto II, BD biosciences) and the data were analyzed by Flowjo software (Flowjo, Ashland, USA). The used antibodies are listed in Table S2 (Supporting Information).

### Mouse Transfusion

The use of mice was approved by the METC (AVD3010020198844). Immunodeficient MISTRG mice (M‐CSF^h/h^ IL‐3/GM‐CSF^h/h^ hSIRPAtg TPO^h/h^ Rag2^–/–^ IL2Rγ^–/–^) were generated as described before (Regeneron;^[^ ^32^ ^]^), and maintained under specific pathogen‐free conditions with continuous enrofloxacin antibiotic treatment in drinking water (Baytril, 0.27 mg mL^−1^; Bayer). Adult mice were administered with clodronate liposomes (LIPOSOMA) at a concentration of 5 mg mL^−1^ to deplete reminiscent murine phagocyte populations. The dosing regimen involved intravenous (IV) injections of 100 µL per mouse on day ‐4, followed by 50 µL per mouse on days −1, 1, and 3. Each treatment group consisted of three mice, with one additional mouse serving as the clodronate untreated control. At day 0 the mice received an IV transfusion of 150 µL per mouse, containing 180 × 10^6^ filter‐purified iRETs or nERYs. Samples of murine peripheral blood (collected both pre‐ and post‐transfusion), as well as spleen, liver, were collected for flow cytometry and ImageStream analysis. Samples were stained with CD235/CD71. The treatment protocol and experimental timeline are illustrated in Figure 7A.

### ImageStream and AI Amnis

RBCs were fixed with glutaraldehyde solution (GA (0.4%) in HEPES buffer, 20 min), stained for CD235/CD71 marker combinations (Table S2, Supporting Information), imaged by ImageStream (Amnis), and analyzed using IDEAS 6.3 software. Amnis AI software in combination with ImageStream was used and trained to classify RBC shapes.

### Statistical Tests

Statistical analyses were performed using GraphPad Prism 9.1.1 software, with t‐tests or ANOVA used as applicable. Test specifics, confidence intervals (CI), Alpha, and sample sizes (*n* numbers) are provided in the figure captions where relevant. All quantifications are presented as mean ± standard deviation (SD), unless otherwise specified.

## Conflict of Interest

The authors declare no conflict of interest.

## Author Contributions

E.V., E.vdA., and M.vL. performed conceptualization. E.V. performed data curation and wrote the original draft. E.V. and E.B. performed formal analysis. E.vdA. and M.vL. performed funding acquisition: E.V., R.R., A.C., R.P., E.B., K.F., J.K., A.L., B.J.G., and M.K. performed investigation. E.V. and E.vdA. performed the methodology. E.V., J.K., and E.vdA. performed project administration. E.V., R.R., A.C., R.P., R.F., and E.vdA. performed resources. E.B. performed software. E.vdA., D.A., and E.V. performed supervision. E.V., E.vdA., M.vL., and K.F. performed visualization. E.V., E.vdA., R.R., A.C., R.P., E.B., K.F., J.K., A.L., R.F., D.A., M.vL., E.vdA., B.J.G., and M.K. wrote, reviewed, and edited.

## Supporting information



Supporting Information

## Data Availability

All data are available in the main text or the supplementary materials.
