# Supplementary material for: Large‐Scale Production of Transfusion‐Ready Red Blood Cells From Induced Pluripotent Stem Cells
Source: Adv Sci (Weinh). 2025 Jul 12;12(38):e04725. doi: 10.1002/advs.202504725 (PMC12520481; doi:10.1002/advs.202504725)
Supplement: Supplementary file 1 — Supporting Information [file ADVS-12-e04725-s001.docx]

Supporting Information

Large-scale production of transfusion-ready red blood cells from induced pluripotent stem cells

Eszter Varga^1^*, Eelke Brandsma^1^, Brenda E. Juarez-Garza^2^, Renuka P.E. Ramlal^1^, Julien J. Karrich^1^, Adrien Laurent^1^, Athina Chavli^1^, Ruthmila Paskel^1^, Kerly Fu^1^, Richard A. Flavell^3,4^, Marieke von Lindern^1^, Derk Amsen^1^, Marieke E. Klijn^2^, Emile van den Akker^1^*


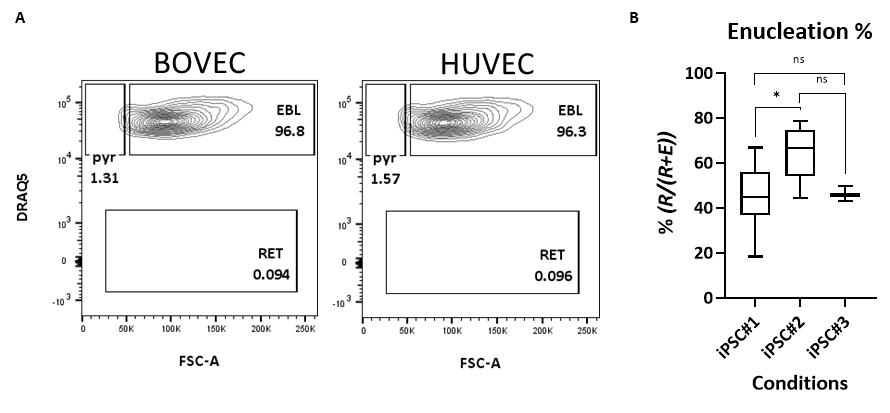


Figure S1.

**Enucleation of iRBCs (connection to Figure 1).** (**A**) Enucleation potential of 2D-iRBCs on feeder layer by flow cytometry plots of CD235+ populations depicting DRAQ5- RET%; DRAQ5+ pyrenocytes% and EBL%. (**B**) Enucleation% of 3D-iRBCs represented per cell lines, based on flow cytometry calculated by RET% / (RET% + EBL%), n≥3, unpaired Mann-Whitney test, 95 % CI.


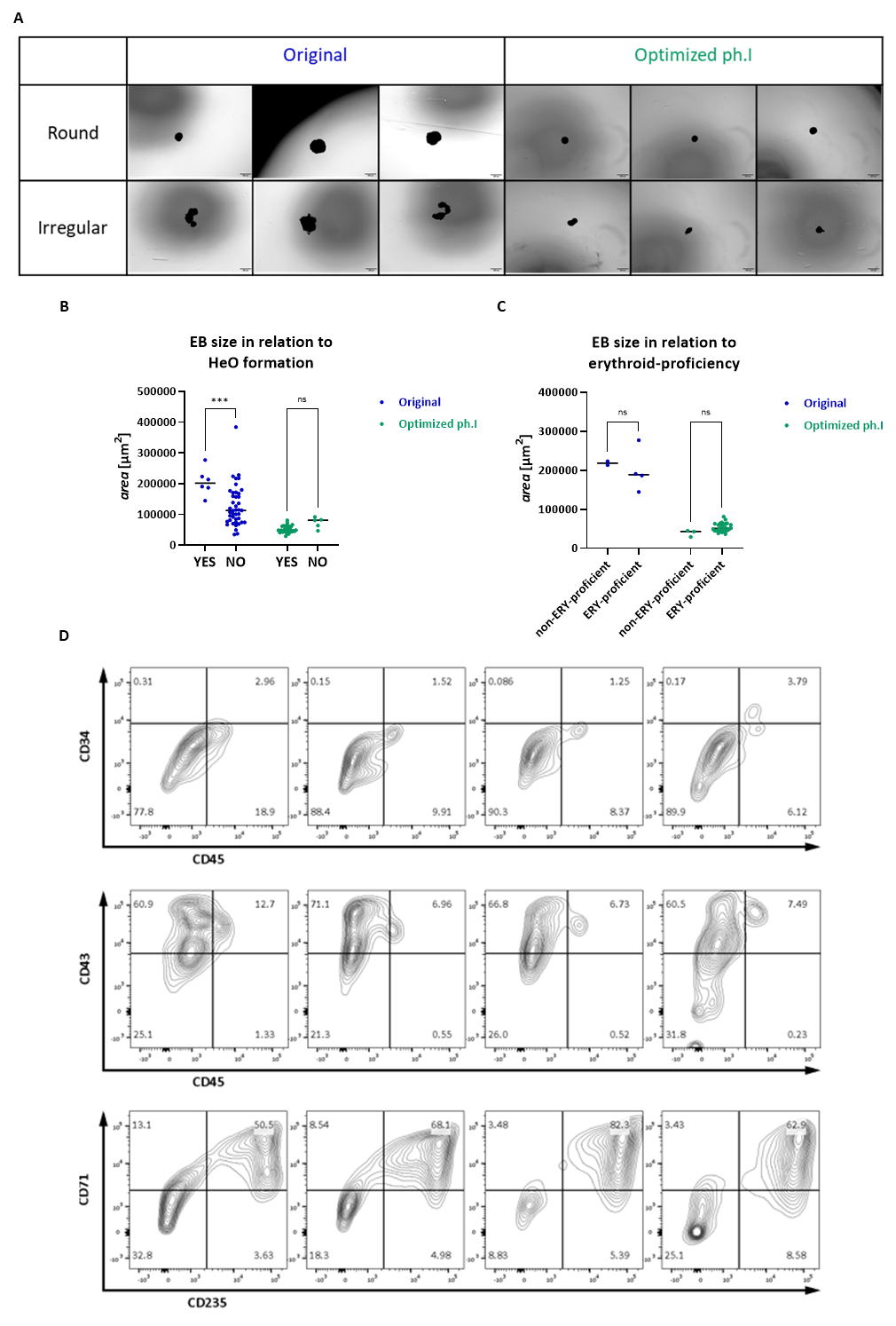
Figure S2.

**Optimized EB formation under dynamic culture condition enhances HeO formation but does not markedly alter hematopoietic and erythroid marker expression patterns (connection to Figure 2).** (**A**) Morphology of EBs generated by the original and dynamic (Ph.I) system. (**B**) HeO formation in relation to EB size (YES: HeOs formed, NO: HeOs did not form), n≥36, Ṧidak’s multiple comparison, Alpha = 0,05, 95 % CI. (**C**) EB size in relation to proficiency n≥6, Ṧidak’s multiple comparison, Alpha = 0,05, 95 % CI (right). (**D**) Weekly marker expression pattern of HSPC using flow cytometry, with representative flow plots.


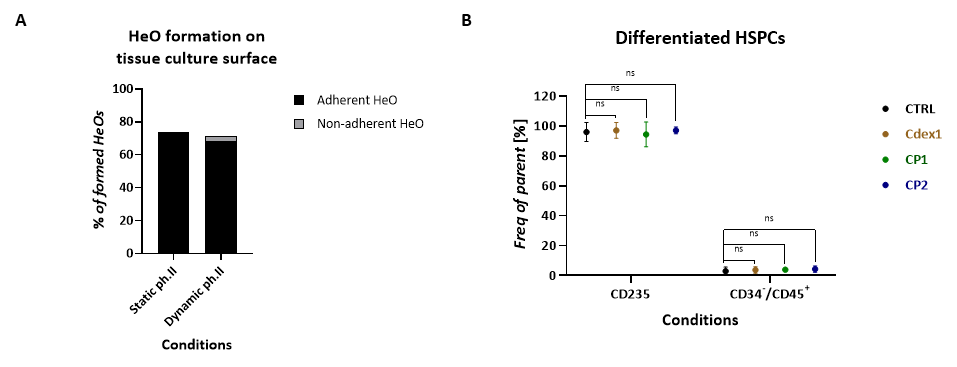


Figure S3.

**Erythroid-proficient HeO formation under dynamic culture condition (connection to Figure 3).** (**A**) Adherence to tissue culture surface in relation to HeO formation under dynamic culture condition, n=35. (**B**) Erythroid purity of terminally differentiated (D14-18) HSPCs derived on MC-mediated HeOs, measured by flow cytometry, n≥6, Dunnett’s multiple comparison, Alpha = 0,05, 95 % CI.


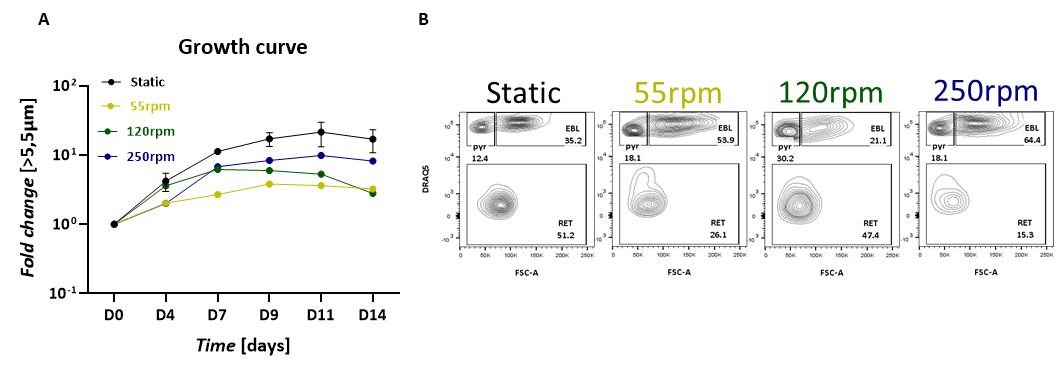
Figure S4.

**Erythroid expansion and maturation can occur under dynamic culture condition (connection to Figure 4).** (**A**) Expansion potential of differentiating HSPCs using different mixing speeds. Static sample conditions are shown with mean and SEM. (**B**) Enucleation potential of the terminally differentiated HSPCs under dynamic culturing, shown by flow cytometry using CD235/CD71 and DRAQ5 staining.


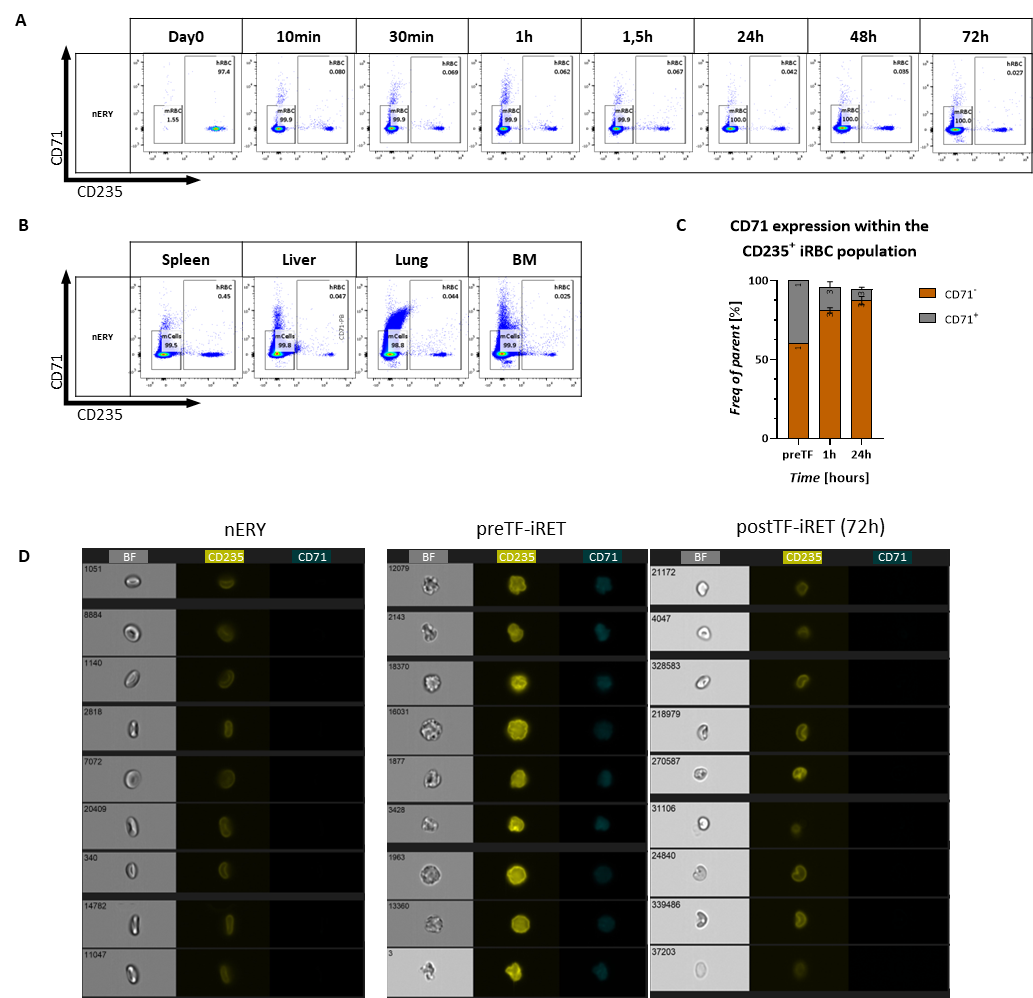


Figure S5.

**Transfusion of human nERYs and iRETs into MISTRG mouse model (connection to Figure 7)**. (**A**) Initial destruction determination performed using human nERYs transfusions into MISTRG mice. (**B**) Distribution of transfused human nERYs in mouse organs. (**C**) Quantification of CD71 marker expression in iRETs by flow cytometry before and after transfusions. (**D**) Morphology and erythroid marker expression of hRBCs pre- and post- transfusions depicted by ImageStream.

Table S1.

**iPSC to RBC differentiation publication list.**

| **Year** | **Cell**  **type** | **Method of initiation** | **Initiation growth factors** | **Av. enucleation %** | | **Reference** |
| --- | --- | --- | --- | --- | --- | --- |
|  |  |  |  | on-feeder | feeder-free |  |
| 2004 | ESC | Directed EB | bFGF, VEGF, BMP4, SCF, FLT3, IL3, IL6, G-CSF, EPO | N/A | | [56] |
| 2006 | ESC | Directed EB | bFGF, VEGF | N/A | | [57] |
| 2006 | ESC | Monolayer co-culture | non | 0 | - | [58] |
| 2008 | ESC | Monolayer co-culture | non | 6,5 [±6,7] | - | [59] |
| 2008 | ESC | Monolayer co-culture | non | N/A | | [60] |
| 2008 | ESC | Directed EB | bFGF, VEGF, BMP4, SCF, FLT3, TPO | 40 [±17] | 10-30 | [61] |
| 2010 | ESC | Directed EB | VEGF, BMP4, SCF, FLT3, IL3, IL6, TPO, EPO | - | 52-66 | [62] |
|  | iPSC |  |  | - | 4-10 |  |
| 2011 | ESC/iPSC | Monolayer co-culture | non | N/A | | [63] |
| 2011 | ESC/iPSC | Monolayer co-culture | non | 2-10 | 0 | [64] |
| 2012 | iPSC | Directed EB | VEGF, BMP4, SCF, FLT3, IL3, IL6, TPO, EPO | - | 20-26 | [65] |
| 2014 | iPSC | Monolayer co-culture | non | - | 10-15 | [66] |
| 2015 | ESC | Directed EB | VEGF, BMP4, SCF, FLT3, IL3, IL6, TPO, EPO | - | 7,9 [±5,3] | [67] |
|  | iPSC |  |  | - | 20,7-28,4 |  |
| 2015 | iPSC | Directed EB | bFGF, VEGF, BMP4, SCF | - | 0,2-4,7 | [68] |
| 2015 | ESC | Directed EB | VEGF, BMP4, SCF, FLT3, IL3, IL6, EPO | - | 42-65 | [69] |
| 2015 | ESC/iPSC | Directed EB | bFGF, BMP4 | N/A | | [70] |
| 2016 | ESC/iPSC | Monolayer co-culture | VEGF | N/A | | [71] |
| 2016 | ESC | Directed microcarrier-EB | bFGF, VEGF, BMP4 | 25 | - | [72] |
| 2016 | ESC | Directed-EB | VEGF, BMP4, Wnt3a, ActivinA, GSK-3β | N/A | | [73] |
| 2016 | ESC/iPSC | Directed EB | VEGF, BMP4, Wnt3a, ActivinA, GSK-3β or A-A014418 | - | 10 | [74] |
| 2016 | ESC/iPSC | Monolayer co-culture | bFGF | N/A | | [75] |
| 2017 | ESC/iPSC | Monolayer co-culture | VEGF | N/A | | [76] |
| 2017 | iPSC | Directed monolayer | ActivinA, BMP4, bFGF | 10 | - | [77] |
| 2017 | iPSC | Directed EB | VEGF, BMP4, Wnt3a, ActivinA, GSK-3β or A-A014418 | - | 2-6 | [78] |
| 2018 | iPSC | Directed monolayer | VEGF, Wnt3a, Matrigel | N/A | | [79] |
| 2018 | iPSC | Directed monolayer | bFGF, VEGF, BMP4 | - | 5 | [27] |
| 2018 | iPSC | Directed microcarrier-EB | bFGF, VEGF, BMP4, ActivinA, CHIR | 28-40 | - | [80] |
| 2019 | iPSC | Directed monolayer/EB | VEGF, BMP4, Wnt3a, ActivinA, GSK-3β | 1,5 | 0,8 | [81] |
| 2019 | iPSC | Directed monolayer | bFGF, VEGF, BMP4, CHIR, SB | - | 41,8±1,1 | [82] |
| 2019 | iPSC | Spontaneous EB | non | - | 44±15,1 | [24] |
| 2019 | iPSC | Directed monolayer | VEGF, BMP4, Wnt3a, Wnt5a, ActivinA, GSK-3β | N/A | | [83] |
| 2020 | ESC/iPSC | Directed EB | VEGF, BMP4, Wnt3a, ActivinA, GSK-3β | N/A | | [84] |
| 2021 | iPSC | Directed microcarrier-EB | bFGF, VEGF, BMP4, ActivinA, CHIR | 18-59,3 | 6,2–6,42 | [26] |
| 2022 | iPSC | Directed monolayer | Stemdiff Hematopoietic media A | - | 13 | [49] |
| 2022 | iPSC | Directed microcarrier-EB | bFGF, VEGF, BMP4, ActivinA, CHIR | 18-30 | 8 | [25] |
| 2022 | iPSC | Spontaneous EB | non | N/A | | [31] |
| 2022 | ESC/iPSC | Directed EB | bFGF, BMP4, Wnt3a, ActivinA | - | 0 | [85] |
| 2023 | iPSC | Directed EB | bFGF, VEGF, BMP4, IWP2 | - | 0-10 | [86] |
| 2023 | ESC | Directed monolayer | VEGF, BMP4, CHIR, | N/A | | [87] |
| 2024 | iPSC | Directed EB | BMP4, CHIR, SB | N/A | | [88] |

Table S2.

**Antibody list.**

| **Antibody** | **Company** | **Cat. Nr.** |
| --- | --- | --- |
| **Hematopoietic marker expression** | | |
| **CD71** | MACS | 130-101-631 |
| **CD235** | Acris | DM066R |
| **DRAQ5** | Abcam | 108410 |
| **CD34** | Biolegend | 343206 |
| **CD45** | BD Biosciences | 348815 |
| **CD43** | Biolegend | 315203 |
| **CD44** | eBioscience | 17-0441-81 |
| **CD49d** | BD Biosciences | 565277 |
| **Blood group phenotype** | | |
| **Anti A** | NHS B&T | 9434CS |
| **Anti B** | NHS B&T | 9432CS |
| **Anti D** | NHS B&T | 9433CS |
| **Anti C** | Sanquin | K1195 |
| **Anti c** | Sanquin | K1196 |
| **Anti E** | Sanquin | K1191 |
| **Anti e** | Sanquin | K1205 |
| **Anti K** | Sanquin | K1199 |
| **Anti k** | Essange | 11305 |
| **Anti M** | Sanquin | K1302 |
| **Anti N** | NHS B&T | 9401CS |
| **Anti S** | Sanquin | K1304 |
| **Anti s** | Sanquin | K1343 |
| **Anti Fy-a** | Essange | 12305 |
| **Anti Fy-b** | Essange | 13305 |
| **Goat Anti-Human IgG - AF647** | Invitrogen | A-21445 |
| **Goat Anti-Human IgM - AF647** | Southern Biotech | 2022-31 |
| **Goat Anti-Mouse Ig - APC** | BD Biosciences | 550826 |

**References**

[56] Cerdan, C., Rouleau, A., and Bhatia, M. (2004) VEGF-A165 augments erythropoietic development from human embryonic stem cells. *Blood*, **103** (7), 2504–12.

[57] Chang, K.-H., Nelson, A.M., Cao, H., Wang, L., Nakamoto, B., Ware, C.B., and Papayannopoulou, T. (2006) Definitive-like erythroid cells derived from human embryonic stem cells coexpress high levels of embryonic and fetal globins with little or no adult globin. *Blood*, **108** (5), 1515–23.

[58] Olivier, E.N., Qiu, C., Velho, M., Hirsch, R.E., and Bouhassira, E.E. (2006) Large-scale production of embryonic red blood cells from human embryonic stem cells. *Exp Hematol*, **34** (12), 1635–42.

[59] Qiu, C., Olivier, E.N., Velho, M., and Bouhassira, E.E. (2008) Globin switches in yolk sac-like primitive and fetal-like definitive red blood cells produced from human embryonic stem cells. *Blood*, **111** (4), 2400–8.

[60] Ma, F., Ebihara, Y., Umeda, K., Sakai, H., Hanada, S., Zhang, H., Zaike, Y., Tsuchida, E., Nakahata, T., Nakauchi, H., and Tsuji, K. (2008) Generation of functional erythrocytes from human embryonic stem cell-derived definitive hematopoiesis. *Proc Natl Acad Sci U S A*, **105** (35), 13087–92.

[61] Lu, S.-J., Feng, Q., Park, J.S., Vida, L., Lee, B.-S., Strausbauch, M., Wettstein, P.J., Honig, G.R., and Lanza, R. (2008) Biologic properties and enucleation of red blood cells from human embryonic stem cells. *Blood*, **112** (12), 4475–84.

[62] Lapillonne, H., Kobari, L., Mazurier, C., Tropel, P., Giarratana, M.-C., Zanella-Cleon, I., Kiger, L., Wattenhofer-Donzé, M., Puccio, H., Hebert, N., Francina, A., Andreu, G., Viville, S., and Douay, L. (2010) Red blood cell generation from human induced pluripotent stem cells: perspectives for transfusion medicine. *Haematologica*, **95** (10), 1651–9.

[63] Chang, C.-J., Mitra, K., Koya, M., Velho, M., Desprat, R., Lenz, J., and Bouhassira, E.E. (2011) Production of embryonic and fetal-like red blood cells from human induced pluripotent stem cells. *PLoS One*, **6** (10), e25761.

[64] Dias, J., Gumenyuk, M., Kang, H., Vodyanik, M., Yu, J., Thomson, J.A., and Slukvin, I.I. (2011) Generation of red blood cells from human induced pluripotent stem cells. *Stem Cells Dev*, **20** (9), 1639–47.

[65] Kobari, L., Yates, F., Oudrhiri, N., Francina, A., Kiger, L., Mazurier, C., Rouzbeh, S., El-Nemer, W., Hebert, N., Giarratana, M.-C., François, S., Chapel, A., Lapillonne, H., Luton, D., Bennaceur-Griscelli, A., and Douay, L. (2012) Human induced pluripotent stem cells can reach complete terminal maturation: *in vivo* and *in vitro* evidence in the erythropoietic differentiation model. *Haematologica*, **97** (12), 1795–803.

[66] Trakarnsanga, K., Wilson, M.C., Griffiths, R.E., Toye, A.M., Carpenter, L., Heesom, K.J., Parsons, S.F., Anstee, D.J., and Frayne, J. (2014) Qualitative and quantitative comparison of the proteome of erythroid cells differentiated from human iPSCs and adult erythroid cells by multiplex TMT labelling and nanoLC-MS/MS. *PLoS One*, **9** (7), e100874.

[67] Dorn, I., Klich, K., Arauzo-Bravo, M.J., Radstaak, M., Santourlidis, S., Ghanjati, F., Radke, T.F., Psathaki, O.E., Hargus, G., Kramer, J., Einhaus, M., Kim, J.B., Kögler, G., Wernet, P., Schöler, H.R., Schlenke, P., and Zaehres, H. (2015) Erythroid differentiation of human induced pluripotent stem cells is independent of donor cell type of origin. *Haematologica*, **100** (1), 32–41.

[68] Huang, X., Wang, Y., Yan, W., Smith, C., Ye, Z., Wang, J., Gao, Y., Mendelsohn, L., and Cheng, L. (2015) Production of Gene-Corrected Adult Beta Globin Protein in Human Erythrocytes Differentiated from Patient iPSCs After Genome Editing of the Sickle Point Mutation. *Stem Cells*, **33** (5), 1470–9.

[69] Rouzbeh, S., Kobari, L., Cambot, M., Mazurier, C., Hebert, N., Faussat, A.-M., Durand, C., Douay, L., and Lapillonne, H. (2015) Molecular signature of erythroblast enucleation in human embryonic stem cells. *Stem Cells*, **33** (8), 2431–41.

[70] Ganji, F., Abroun, S., Baharvand, H., Aghdami, N., and Ebrahimi, M. (2015) Differentiation potential of o bombay human-induced pluripotent stem cells and human embryonic stem cells into fetal erythroid-like cells. *Cell J*, **16** (4), 426–39.

[71] Fujita, A., Uchida, N., Haro-Mora, J.J., Winkler, T., and Tisdale, J. (2016) β-Globin-Expressing Definitive Erythroid Progenitor Cells Generated from Embryonic and Induced Pluripotent Stem Cell-Derived Sacs. *Stem Cells*, **34** (6), 1541–52.

[72] Sivalingam, J., Lam, A.T.-L., Chen, H.Y., Yang, B.X., Chen, A.K.-L., Reuveny, S., Loh, Y.-H., and Oh, S.K.-W. (2016) Superior Red Blood Cell Generation from Human Pluripotent Stem Cells Through a Novel Microcarrier-Based Embryoid Body Platform. *Tissue Eng Part C Methods*, **22** (8), 765–80.

[73] Jackson, M., Ma, R., Taylor, A.H., Axton, R.A., Easterbrook, J., Kydonaki, M., Olivier, E., Marenah, L., Stanley, E.G., Elefanty, A.G., Mountford, J.C., and Forrester, L.M. (2016) Enforced Expression of HOXB4 in Human Embryonic Stem Cells Enhances the Production of Hematopoietic Progenitors but Has No Effect on the Maturation of Red Blood Cells. *Stem Cells Transl Med*, **5** (8), 981–90.

[74] Olivier, E.N., Marenah, L., McCahill, A., Condie, A., Cowan, S., and Mountford, J.C. (2016) High-Efficiency Serum-Free Feeder-Free Erythroid Differentiation of Human Pluripotent Stem Cells Using Small Molecules. *Stem Cells Transl Med*, **5** (10), 1394–1405.

[75] Mao, B., Huang, S., Lu, X., Sun, W., Zhou, Y., Pan, X., Yu, J., Lai, M., Chen, B., Zhou, Q., Mao, S., Bian, G., Zhou, J., Nakahata, T., and Ma, F. (2016) Early Development of Definitive Erythroblasts from Human Pluripotent Stem Cells Defined by Expression of Glycophorin A/CD235a, CD34, and CD36. *Stem Cell Reports*, **7** (5), 869–883.

[76] Uchida, N., Haro-Mora, J.J., Fujita, A., Lee, D.-Y., Winkler, T., Hsieh, M.M., and Tisdale, J.F. (2017) Efficient Generation of β-Globin-Expressing Erythroid Cells Using Stromal Cell-Derived Induced Pluripotent Stem Cells from Patients with Sickle Cell Disease. *Stem Cells*, **35** (3), 586–596.

[77] Razaq, M.A., Taylor, S., Roberts, D.J., and Carpenter, L. (2017) A molecular roadmap of definitive erythropoiesis from human induced pluripotent stem cells. *Br J Haematol*, **176** (6), 971–983.

[78] Yang, C.-T., Ma, R., Axton, R.A., Jackson, M., Taylor, A.H., Fidanza, A., Marenah, L., Frayne, J., Mountford, J.C., and Forrester, L.M. (2017) Activation of KLF1 Enhances the Differentiation and Maturation of Red Blood Cells from Human Pluripotent Stem Cells. *Stem Cells*, **35** (4), 886–897.

[79] Vanuytsel, K., Matte, T., Leung, A., Naing, Z.H., Morrison, T., Chui, D.H.K., Steinberg, M.H., and Murphy, G.J. (2018) Induced pluripotent stem cell-based mapping of β-globin expression throughout human erythropoietic development. *Blood Adv*, **2** (15), 1998–2011.

[80] Sivalingam, J., Chen, H.Y., Yang, B.-X., Lim, Z.R., Lam, A.T.L., Woo, T.L., Chen, A.K.-L., Reuveny, S., Loh, Y.-H., and Oh, S.K.-W. (2018) Improved erythroid differentiation of multiple human pluripotent stem cell lines in microcarrier culture by modulation of Wnt/β-Catenin signaling. *Haematologica*, **103** (7), e279–e283.

[81] Lopez-Yrigoyen, M., Yang, C.-T., Fidanza, A., Cassetta, L., Taylor, A.H., McCahill, A., Sellink, E., von Lindern, M., van den Akker, E., Mountford, J.C., Pollard, J.W., and Forrester, L.M. (2019) Genetic programming of macrophages generates an *in vitro* model for the human erythroid island niche. *Nat Commun*, **10** (1), 881.

[82] Olivier, E.N., Zhang, S., Yan, Z., Suzuka, S., Roberts, K., Wang, K., and Bouhassira, E.E. (2019) PSC-RED and MNC-RED: Albumin-free and low-transferrin robust erythroid differentiation protocols to produce human enucleated red blood cells. *Exp Hematol*, **75**, 31-52.e15.

[83] Ruiz, J.P., Chen, G., Haro Mora, J.J., Keyvanfar, K., Liu, C., Zou, J., Beers, J., Bloomer, H., Qanash, H., Uchida, N., Tisdale, J.F., Boehm, M., and Larochelle, A. (2019) Robust generation of erythroid and multilineage hematopoietic progenitors from human iPSCs using a scalable monolayer culture system. *Stem Cell Res*, **41**, 101600.

[84] Park, Y.J., Jeon, S.-H., Kim, H.-K., Suh, E.J., Choi, S.J., Kim, S., and Kim, H.O. (2020) Human induced pluripotent stem cell line banking for the production of rare blood type erythrocytes. *J Transl Med*, **18** (1), 236.

[85] Roh, J., Kim, S., Cheong, J.-W., Jeon, S.-H., Kim, H.-K., Kim, M.J., and Kim, H.O. (2022) Erythroid Differentiation of Induced Pluripotent Stem Cells Co-cultured with OP9 Cells for Diagnostic Purposes. *Ann Lab Med*, **42** (4), 457–466.

[86] Cho, Y.K., Kim, H.-K., Kwon, S.S., Jeon, S.-H., Cheong, J.-W., Nam, K.T., Kim, H.-S., Kim, S., and Kim, H.O. (2023) *In vitro* erythrocyte production using human-induced pluripotent stem cells: determining the best hematopoietic stem cell sources. *Stem Cell Res Ther*, **14** (1), 106.

[87] Jeon, S.-B., Koh, H., Han, A.-R., Kim, J., Lee, S., Lee, J.-H., Im, S.-S., Yoon, Y.-S., Lee, J.-H., and Lee, J.Y. (2023) Ferric citrate and apo-transferrin enable erythroblast maturation with β-globin from hemogenic endothelium. *NPJ Regen Med*, **8** (1), 46.

[88] Pavani, G., Klein, J.G., Nations, C.C., Sussman, J.H., Tan, K., An, H.H., Abdulmalik, O., Thom, C.S., Gearhart, P.A., Willett, C.M., Maguire, J.A., Chou, S.T., French, D.L., and Gadue, P. (2024) Modeling primitive and definitive erythropoiesis with induced pluripotent stem cells. *Blood Adv*, **8** (6), 1449–1463.
